# Beyond Language Scores: How Language Exposure Informs Assessment of Nonword Repetition, Vocabulary and Narrative Macrostructure in Bilingual Turkish/Swedish Children with and without Developmental Language Disorder

**DOI:** 10.3390/children11060704

**Published:** 2024-06-07

**Authors:** Linnéa Öberg, Ute Bohnacker

**Affiliations:** Department of Linguistics & Philology, Uppsala University, P.O. Box 635, SE-75126 Uppsala, Sweden; linnea.oberg@lingfil.uu.se

**Keywords:** bilingual children, Developmental Language Disorder (DLD), language exposure, narrative macrostructure, nonword repetition, Swedish, Turkish, vocabulary size

## Abstract

As in many other countries, baseline data concerning the linguistic development of bilingual children in Sweden are lacking, and suitable methods for identifying developmental language disorder (DLD) in bilinguals are lacking as well. This study presents reference data from 108 typically developing (TD) Turkish/Swedish-speaking children aged 4;0–8;1, for a range of language tasks developed specifically for the assessment of bilinguals (LITMUS test battery, COST Action IS0804). We report on different types of nonword repetition (NWR) tasks (language-specific and language-independent), receptive and expressive vocabulary (Cross-Linguistic Lexical Tasks, CLTs), and narrative macrostructure comprehension and production (Multilingual Assessment Instrument for Narratives, MAIN) in Turkish, the children’s home language, and in Swedish, the language of schooling and society. Performance was investigated in relation to age, language exposure, type of task, and (for NWR and narratives) vocabulary size. There was a positive development with age for all tasks, but effects of language exposure and vocabulary size differed between tasks. Six bilingual Turkish/Swedish children with DLD were individually compared to the TD children. TD/DLD performance overlapped substantially, particularly for NWR, and more so for the production than the comprehension tasks. Surprisingly, the discriminatory potential was poor for both language-specific and language-independent NWR. DLD case studies underscored the importance of interpreting language scores in relation to exposure history, and the need for an increased emphasis on functional language skills as reported by parents and teachers when assessing and diagnosing DLD in bilinguals.

## 1. Introduction

A large proportion of children in Sweden today are growing up multilingually, with a home language other than the language of society and schooling. A total of 28.3% of all children aged 7 to 15 in Sweden are entitled to heritage language education, which means that these children speak a home language other than, or in addition to, Swedish (National Agency for Education 2023 [1]), and in many urban areas, the proportion is more than twice as high. In such linguistically and culturally diverse settings, it is often a challenge to identify developmental language and learning disorders, as the children’s majority-language skills are still developing, and effective language assessment materials and methods for multilinguals are few and far between. Norm-referenced tests are mostly standardized on monolingual child populations, and in countries with a relatively small population such as Sweden (10 million), proper norms may be lacking altogether; alternatively, existing tests frequently over-identify developmental language disorder (DLD)^1^ in multilinguals, which casts doubt on their clinical utility. For instance, Andersson et al. (2019 [5]) found that 80% of the 224 bilingual 7-to-8-year-olds in their sample performed more than 1 SD below the normative monolingual mean on the Swedish version of a widely used clinical test, the Clinical Evaluation of Language Fundamentals (CELF-4).

In response to similar diagnostic test shortcomings also in other countries, and because of a general lack of suitable (and affordable) assessment materials, the research network COST Action IS0804 *Language impairment in a multilingual society: Linguistic patterns and the road to assessment* was founded (https://www.bi-sli.org/). From 2009 to 2013, a network of (mainly European) researchers in linguistics, speech-language therapy and developmental psychology collaborated in basic research and developed comparable language assessment materials and methods/tasks for many different languages, which expressly were not just translations of a test that had first been developed for one particular language (such as English). After the official end of the COST Action, many of the researchers continued to collaborate, and work with the Action materials has branched out into other parts of the world. Thus, a battery of tasks has been gathered under the umbrella of LITMUS (Language impairment testing in multilingual settings, Armon-Lotem et al., 2015 [6]). The Action and its spinoffs have been most productive, and new language versions of the LITMUS tasks are continually being developed. Since several tasks have been made freely available to other researchers and practitioners, their use has spiraled in many parts of the world.

The present paper investigates how bilingual Turkish/Swedish children with and without DLD fare on the LITMUS tasks in three domains: nonword repetition, cross-linguistic lexical tasks, and narrative macrostructure. They were developed with a view to provide less-biased methods of language assessment than the existing standardized language tests for bilingual children of a wide variety of language combinations. The rationale behind them can be captured as follows.

In *nonword repetition* (NWR), children are asked to repeat phonological forms that are not real words and that they have not heard before. NWR draws on phonological processing and cognitive-perceptual mechanisms.^2^ A deficit in verbal short-term memory is commonly associated with DLD (Gathercole & Baddeley 1990 [8]). Poor NWR performance is regarded as a clinical marker of DLD, as many studies have found that (monolingual) children with a DLD diagnosis show impaired NWR performance compared to TD peers (e.g., Chiat 2015 [9]) across languages, including for Turkish (Topbaş et al., 2014 [10]) and Swedish (Kalnak et al., 2014 [11]), the two languages of the present study. Impaired NWR performance has been documented for bilingual children with DLD as well, even though the evidence here is not as clear-cut as for monolinguals (Ortiz 2021 [12]; Schwob et al., 2021 [13]). There are no studies of NWR in a Swedish/Turkish context, and only one published study on NWR in bilinguals in Sweden (Öberg & Bohnacker 2022 [14], on Arabic/Swedish, see Section 2.1). Internationally, NWR has been promoted as a valid and diagnostically useful tool for the clinical assessment of bilingual children (Antonijevic-Elliott et al., 2020 [15]; Boerma et al., 2015 [16]; Boerma & Blom 2021 [17]; Chiat 2015 [9]; Paradis et al., 2021, p. 330 [18]; Sorenson Duncan & Paradis 2016 [19]; Thordardottir 2015, p. 348 [20]; Thordardottir & Brandeker 2013 [21]). Bilinguals are assumed to be able to transfer the phonological processing skills they have acquired in any language; thus, the performance on an NWR task should remain largely unaffected by limited exposure and/or language knowledge, especially when the nonword items are quasi-universal, i.e., (relatively) independent of language-specific traits. On such NWR tasks, TD bilinguals are predicted to be able to perform on a par with TD monolinguals, and better than DLD children. (For studies on this, see Section 2.1 below.) Moreover, since phonological processing skills are assumed to be transferrable between languages, it is often deemed sufficient to assess NWR in bilingual children in only one of their languages, especially when quasi-universal nonword items are used (Boerma & Blom 2017 [22], 2021 [17]; Dollaghan & Campbell 1998 [23]; Thordardottir & Brandeker 2013:, p. 3 [21]).^3^ The LITMUS battery includes several NWR tasks that are more or less language-(in)dependent, with items constructed according to certain principles (https://www.bi-sli.org/nonword-repetition, see also Section 2.1 and Section 4.2.1).

Moving on from nonwords to real words (*vocabulary*), delayed and impaired lexical abilities are known to be one of the earliest indicators of DLD for monolingual children (e.g., Leonard 2014, pp. 53–57 [3]; Rice & Hoffman 2015 [26]; Trauner et al., 2000 [27]). This appears to hold for DLD in bilinguals as well; however, studies on the clinical utility of vocabulary assessments have come to divergent conclusions. Vocabulary development is heavily dependent on language input and exposure, and as the amount of exposure is necessarily divided between two languages in bilingual children, TD bilinguals may show lexical gaps and depressed vocabulary scores compared to monolingual peers (Oller & Eilers 2002 [28]; Thordardottir 2011 [29]). Bilinguals are frequently tested in one language only (typically in the majority or societal language, where vocabulary tests are available), even though this procedure does not fairly represent their full lexical competence. Comparable, parallel vocabulary tasks could be used to clinically assess the two languages of bilinguals, but for most language combinations such tasks do not (yet) exist. As part of the COST Action IS0804, cross-linguistic lexical tasks (CLTs) were developed using a multi-language parallel task-construction procedure (for details see Haman et al., 2015 [30], https://multilada.pl/en/projects/clt/), in order to provide fully comparable assessment tools for the comprehension and production of nouns and verbs in different languages and language combinations (see Section 2.2). The CLT developers assume that when lexical deficits are identified in both languages of a bilingual child, this is a warning sign for DLD (Haman et al., 2015, p. 198 [30]; Haman & Łuniewska 2013 [31]). There are, however, surprisingly few published studies that explore the diagnostic utility of CLT vocabulary assessment in bilingual children (see Section 2.2).

*Narrative macrostructure* is another domain that has been promoted as particularly suitable for the language assessment of bilingual children (Govindarajan & Paradis 2019 [32]; Paradis et al., 2013 [33], 2021, p. 330 [18]). Macrostructural skills include the ability to identify causal, hierarchical and thematic relations between events, resulting in an understanding of the overall structure of a story (so-called story grammar) and the ability to produce a coherent story (Stein & Glenn 1979 [34]). Children with DLD are frequently described as performing poorly on narrative tasks, across languages (Mäkinen 2014 [35]; Merritt & Liles 1987 [36]; Soodla & Kikas 2010 [37]). Of course, narrative skills involve many different aspects of language, some of which are highly language-specific (e.g., vocabulary, tense marking, morphosyntactic encoding of reference), and some may also be culture-specific. However, narrative macrostructure is generally regarded as being largely independent of the structural properties of a language and possibly shared across languages, due to its strong cognitive-general underpinnings (e.g., Berman 2001, pp. 420–426 [38]; Berman & Slobin 1994 [39]; Paradis et al., 2021, pp. 170–171 [18]; Pearson 2002 [40]). This would allow bilinguals to use the macrostructural skills they have acquired in any language and transfer them to another language when narrating or comprehending stories. TD bilinguals should thus be able to advance their macrostructural skills in a new language relatively fast, and tasks probing narrative macrostructure should be able to discriminate between bilingual TD and DLD. Only a few studies have evaluated the diagnostic validity of narrative tasks for children learning two or more languages (see Section 2.3). The Multilingual Assessment Instrument for Narratives (MAIN, Gagarina et al., 2019 [41]), https://main.leibniz-zas.de/) assesses both the comprehension and production of macrostructure (see Section 2.3).

Whilst neither macrostructure nor NWR tasks are free of language, the rationale for promoting them as suitable assessment tools for bilinguals is that they have underlying cognitive-general components and tap into skills that are acquired in any language. This may reduce assessment bias against children with limited exposure and different language experiences.

The above LITMUS tasks were developed to support the identification of DLD in bilingual children. However, as bilingual language development is complex, varied, and influenced by a range of child-internal and child-external factors, it is not very likely that we will find a ‘quick fix’, such as the one language task that serves as a good discriminator of DLD; this may also be exacerbated by the heterogeneity of the disorder. We might need a combination (or battery) of tasks. However, it is also possible that the development and use of new language tasks as such will not help us to identify DLD in bilinguals. To interpret the performance on such tasks, we may need substantial background information on the child: reports on language exposure, language use at home and in (pre)school, early developmental milestones, family risk factors, parental and/or teacher concerns, and reports of functional language skills and communication difficulties (Öberg & Bohnacker 2022 [14]; Tuller 2015 [42]). Such information can be gathered via interviews with parents and teachers and/or via questionnaires (e.g., Paradis et al., 2010 [43] for the Canadian ALDEQ; Tuller 2015 [42] for the LITMUS PABIQ).

Since the official end of the COST Action in 2013, hundreds of studies have been carried out that employ one or several of the LITMUS tools, advancing our knowledge about monolingual and/or bilingual child language acquisition. Despite this flurry of papers, books and special issues (e.g., *Applied Psycholinguistics* 37(1), 2016; Armon-Lotem & Grohmann 2021 [44]; Bohnacker & Gagarina 2020b [45]), it is noteworthy that only a small number of publications using the LITMUS language tasks provide reference data for a large enough group of TD bilingual children with the same language combination. Even fewer publications compare the performance of bilingual TD and bilingual DLD children growing up with the same language combination and in comparable settings. Very few studies investigate both of the children’s languages. Exposure and age are not always factored in. Studies that do include bilingual TD and DLD children largely confine themselves to reporting statistically significant differences between the TD and DLD groups. Whilst significant differences between groups may appear promising at first sight, not all studies expound on the finding that the performances of bilingual TD and DLD groups usually show a substantial amount of overlap. At the individual level then, particular language assessment tasks may not be that accurate, and accuracy at the individual level is what determines clinical utility.

The present study presents reference data for more than one hundred bilingual Turkish/Swedish TD 4-to-7-year-olds raised in Sweden for a range of language tasks from the LITMUS battery in both languages, investigating age, exposure factors and vocabulary size as predictors of performance, and comparing the different tasks to each other. The individual performances of a small sample of Turkish/Swedish DLD children are then compared to the TD group.

## 2. Previous Studies

This section provides an overview of existing studies that use the LITMUS NWR or CLT or MAIN with both bilingual TD and bilingual DLD children, and summarizes the main findings from these, as well as some other relevant studies in this domain.

### 2.1. NWR

Several North American studies on the clinical utility of NWR tasks with bilinguals have reported considerable TD/DLD overlap and mixed results regarding their diagnostic accuracy (e.g., Gutiérrez-Clellen & Simon-Cereijido 2010 [46]; Kohnert et al., 2006 [47]; Paradis et al., 2013 [33]; Sorenson Duncan & Paradis 2016 [19]; Windsor et al., 2010 [48]). They largely used language-specific NWR items.

In Europe, the COST Action IS0804 (e.g., Chiat 2015 [9]) put forward the idea that language-independent (quasi-universal) NWR tasks show greater potential to differentiate DLD from TD in bilingual children than language-specific NWR tasks. For instance, in the Netherlands, Boerma et al. (2015 [16]) examined the NWR performance of 120 monolingual and bilingual (early L2 Dutch, mixed L1) children aged 5–6 with and without a diagnosis of DLD. Two different NWR tasks were used, a language-specific one with Dutch-like nonwords, and a language-independent (quasi-universal) one, consisting of 2-to-5-syllable items with simple CV syllable structure, no clusters and quasi-neutral prosody (Chiat 2015 [9]; Chiat & Polišenská 2016 [49]). (This quasi-universal NWR task has since then been renamed and is now called the cross-linguistic NWR task (https://www.bi-sli.org/cl-nonword-repetition). Both NWR tasks distinguished the TD bilingual group (N = 30) from the DLD bilingual group (N = 30), but the quasi-universal NWR task did so with higher sensitivity than the Dutch-specific NWR. There were significant differences between the two groups, but Boerma et al.’s scores of individual TD and DLD children showed a substantial overlap, even though their preselected DLD group was made up of children in special education schools with relatively great educational needs who performed at least 2 SD below the mean on a standardized Dutch test battery (e.g., CELF) or at least 1.5 SD below the mean on two subscales. The L1s of the bilingual children were not assessed; all testing was carried out in Dutch. Boerma and Blom (2017 [22]), investigating roughly the same participant sample with a range of measures, again recommended the language-independent NWR task (Chiat 2015 [9]) as a good diagnostic tool for bilingual children. In a longitudinal follow-up, Boerma and Blom (2021 [17]) retested the same children on the same tasks over the course of two years (from age 5–6 to 7–8). Whilst the NWR results still distinguished the DLD children from the TD children at group level when they were older, classification accuracy was lower than it had been at age 5–6, at below 80%.

A number of other studies emanating from the COST Action have also recommended NWR tasks for diagnostic use, documenting significant differences in the NWR performance of bilingual DLD and bilingual TD children at group level (e.g., Meir 2017 [50], republished in Meir 2021 [51]) for Russian–Hebrew bilinguals (N = 108, age 5;5–6;9); Chilla et al. (2021 [52]) for bilinguals aged 5;5–9;0, with L2 German (N = 56) or L2 French (N = 95); and dos Santos and Ferré (2018 [53]) for bilinguals aged 5;4–8;2 with L2 French (N = 43)).^4^ These studies did not use the same quasi-universal NWR task as Boerma and colleagues, but other NWR tasks from the LITMUS battery, and included both language-specific and language-independent items. While there were significant differences between the TD and DLD groups in these studies, the NWR scores of individual DLD and TD children again showed substantial overlaps. Bilingual DLD sample size was generally small, and despite wide age ranges, the potential effect of age was not investigated.

A recent study of 110 Arabic/Swedish children aged 4–7 with and without a diagnosis of DLD (Öberg & Bohnacker 2022 [14]) investigated the NWR performance on three different NWR tasks, and explored it in relation to age, language exposure, Swedish and Arabic vocabulary size, and NWR item properties (length and complexity). The Arabic/Swedish-speaking children performed a language-specific Swedish NWR task (with word-like items, 2-to-5 syllables, also involving clusters) and two language-independent LITMUS NWR tasks, one being the same as the one used by Boerma and colleagues (2015 [16], 2021 [17]), with simple CV syllable structure in all items (2-to-5 syllables, Chiat 2015 [9]), the other being another language-independent task (1-to-3 syllables, with clusters, dos Santos and Ferré 2018 [53]).^5^ While repetition accuracy varied across these tasks and was influenced by age, vocabulary size and NWR item properties, none of the three NWR tasks, nor any particular type of nonword item, could identify DLD to a satisfactory degree in the Arabic/Swedish bilingual children. Diagnostic utility was poor.

In an as yet unpublished large-scale study (Polišenská et al., 2022 [59]), which combines the datasets of 1742 children from 18 teams spanning 15 countries using similar versions of the same language-independent NWR task (Chiat 2015 [9]), repetition accuracy could not reliably differentiate children with a diagnosis of DLD from their TD peers.

Two recent reviews and meta-analyses of the discriminative function of NWR tasks also report mixed results for bilinguals (Ortiz 2021 [12]; Schwob et al., 2021 [13]). Many of the studies included in the meta-analyses did not reach a diagnostically informative value for language impairment. Both reviews also point to a possible publication bias, where studies reporting positive results concerning the discriminative function of NWR are being favored over studies with null results. The question of the diagnostic utility of NWR tasks with bilingual children is thus still unresolved.

The present paper is the first to explore NWR in a Turkish/Swedish context.

### 2.2. Vocabulary Assessment in Both Languages

When there is a suspicion of DLD, assessment often includes the child’s vocabulary skills (e.g., Peña et al., 2016 [25]). However, since bilinguals have their vocabulary distributed across both languages and since vocabulary size/growth is dependent on exposure, TD bilingual performance on standardized vocabulary tests may vary greatly. Some researchers therefore ascribe low clinical utility to vocabulary assessment tasks when trying to rule in or rule out DLD in bilinguals (e.g., Boerma & Blom 2017 [22]; Paradis et al., 2013 [33]). Other researchers, such as Smolander et al. (2021 [60]), find vocabulary tasks to be clinically useful for the assessment of bilingual children, provided that vocabulary scores are interpreted in relation to language exposure, or provided that the lexical skills in both languages are assessed (e.g., Peña et al., 2016 [25]).

As for the cross-linguistic vocabulary tasks (CLT, Haman et al., 2015 [30]) developed as part of the COST Action IS0804, we only know of two published studies on their diagnostic accuracy in bilinguals. Khoury Aouad Saliby et al., 2017 [61]) compared the performance of 32 TD and 10 DLD bilingual children aged 5;7–7;10 in Lebanon who spoke Lebanese Arabic and French and/or English. Both groups scored near ceiling on Arabic noun comprehension, and also very highly on Arabic verb comprehension. The DLD group did score significantly lower than the TD group on both comprehension and production.^6^ Age and exposure were not factored in. Khoury et al. described the DLD children as having particular problems with the production of verbs.^7^ Despite a visible TD/DLD overlap in the plotted scores, Khoury et al. characterized the CLT as a diagnostically useful tool, stating that “the CLT-LB is reliable in the Lebanese context, as it differentiates Bi-TD from Bi-SLI children” (p. 16).

In another, larger, study also involving Arabic, but this time in a Swedish setting, Öberg and Bohnacker (2022 [14]) used CLTs with 110 Arabic–Swedish-speaking bilinguals aged 4;0–7;11 (99 TD and 11 DLD), both in their L1 Arabic and in their L2 Swedish. The children’s vocabulary scores were strongly influenced by age and exposure. Whilst some of the DLD children scored below their TD peers on either Arabic or Swedish vocabulary or both, others performed on a par with TD peers. In short, the CLT scores could not reliably distinguish between the DLD and TD children. Based on the results of these two studies, it is difficult to predict how other bilingual children will perform on the CLT vocabulary tasks.

In her doctoral thesis, Öztekin (2019 [62]) used a subset of the data of the present study to investigate the CLT scores of Turkish–Swedish-speaking children aged 4;0–8;1. Her comparisons of different age groups (4- vs. 5- vs. 6- vs. 7-year-olds) suggest an effect of age, particularly for the majority-language Swedish, and her longitudinal follow-up study of ten four-year-olds points in the same direction. Öztekin also argued that longer exposure time to Swedish, as well as an estimated daily exposure to Swedish at or above 80%, was associated with higher scores in Swedish. However, Öztekin (2019 [62]) did not employ any multivariate statistics to explore the potential/combined effects of different child-internal and environmental factors on the children’s vocabulary scores. The present study does just this, for both Turkish and Swedish.

### 2.3. Narrative Macrostructure

Prior to the COST Action, few studies investigated the narrative macrostructure of bilingual children in both their languages, or in bilinguals with and without DLD, in part due to a lack of comparable narrative assessment materials across languages. Some (mainly North American) studies that only considered the majority language reported impaired macrostructural production in DLD bilingual children compared to TD bilingual children (e.g., Govindarajan & Paradis 2019 [32]; Paradis et al., 2013 [33]; Rezzonico et al., 2015 [63]). Other studies did not document any significant differences in macrostructure between these groups (e.g., Cleave et al., 2010 [64]; Iluz-Cohen & Walters 2012 [65]). These mixed results may be to do with the use of very different stimulus materials, elicitation modes (retelling vs. story generation) and macrostructural scoring methods. As for the comprehension of narrative macrostructure, which has been found to be impaired in DLD monolinguals (e.g., Bishop & Adams 1992 [66]; Dodwell & Bavin 2008 [67]; Merritt & Liles 1987 [36]), little is known about DLD bilinguals. The launch of the Multilingual Assessment Instrument for Narratives, MAIN, at the final conference of the COST Action (Gagarina et al., 2012 [68]), the subsequent launch of MAIN-Revised (Gagarina et al., 2019 [41]), and its availability in more than 80 languages (https://main.leibniz-zas.de/) has led to exponential growth in the field and may have the potential to unify bilingual narrative research methodologically.^8^ However, published studies that compare the narrative macrostructure skills of bilingual children with and without DLD are still very rare.

Tsimpli et al. (2016 [70]) let Greek-speaking children retell MAIN stories that they had listened to on a computer, including 15 TD and 15 DLD bilinguals (early L2 Greek, mixed L1s, aged 5–11). Results for MAIN story structure were not reported. For story complexity (calculated according to a formula of their own), Tsimpli et al. found no significant difference between the TD and DLD groups. It is difficult to draw any conclusions from this lack of a group difference, since neither exposure nor age (5–11 years) were factored in. The same group of researchers (Peristeri et al., 2020 [71]) also investigated the comprehension of macrostructure in 120 Greek-speaking children, including 30 TD and 30 DLD bilinguals aged 6;0–7;9 (L1 Albanian, and early L2 Greek); the sample overlapped in part with Tsimpli et al. The children answered the MAIN comprehension questions after listening to the story on a computer and retelling it to an experimenter. For this procedure and age range, narrative comprehension scores were high and the means did not significantly differ between the TD and DLD bilingual groups. However, this null result is hard to interpret, since the DLD children were more Greek-dominant than the TD group, according to Peristeri et al.’s measures of exposure and current language use. Only the children’s L2 Greek was assessed.

Boerma et al. (2016 [72]) investigated the comprehension and production of macrostructure with MAIN for 132 monolingual and bilingual children aged 5–6, in Dutch, including 33 TD and 33 DLD bilingual children (early L2 Dutch, mixed L1s); the sample greatly overlapped with the one in Boerma et al. (2015 [16]) described earlier for NWR. Only the children’s L2 Dutch was assessed. As the authors found significant differences between the TD and the DLD groups for macrostructure production, and even more so for the comprehension of macrostructure, they considered MAIN as a diagnostically valid tool for bilingual children. However, classification accuracy in Boerma et al. (2016 [72]) was only around 80%, which is not sufficient for clinical purposes, as the authors acknowledge themselves.^9^ In a study on the same participants, Boerma and Blom (2017 [22]) recommend that MAIN be used together with a language-independent/quasi-universal NWR task and a parental questionnaire to identify DLD in bilingual children. In a longitudinal follow-up that retested the same children on the same tasks, Boerma and Blom (2021 [17]) found that the magnitude of the difference in scores between the TD and DLD groups had shrunk over the course of two years. In fact, by age 7–8, the MAIN comprehension task (after listening to the story) no longer differentiated the TD and DLD groups, due to ceiling effects. Performance on the MAIN comprehension task without prior listening to the story could still differentiate the two groups, though less clearly than at lower ages, and this also held for the MAIN macrostructure production measure. Since the scores of individual DLD and TD children showed substantial overlaps, the question of the diagnostic validity of narrative macrostructure assessment in bilinguals remains open and needs to be researched further.

Öztekin’s (2019 [62]) doctoral thesis includes an investigation of the MAIN comprehension and production tasks for the Turkish/Swedish bilingual participants of the present study. Her comparisons of different age groups (4- vs. 5- vs. 6- vs. 7-year-olds) suggest an effect of age, particularly for the majority-language Swedish. However, Öztekin (2019 [62]) did not use any multivariate statistics to investigate background factors (such as age, length of L2 exposure, current daily L1/L2 exposure, or L1 and L2 vocabulary size) as predictors for narrative performance. The present paper adds this, for both Turkish and Swedish.

## 3. The Present Study: Aims and Research Questions

Although Turkish/Swedish-speaking children are a sizeable minority in Sweden, little is known about their language skills and DLD. There is a general lack of studies on this population, save for Öztekin’s doctoral thesis (2019 [62]). As in many other countries, research on reliable tools for DLD diagnosis in bilingual children is lacking in Sweden.

None of the (aforementioned) LITMUS language tasks are norm-referenced yet, therefore reference data from TD bilinguals are needed for a wide variety of language combinations, including information on task performance in relation to age, exposure and other language measures. Such reference data are a baseline to compare the performance of bilingual children with a DLD diagnosis against, not just at group level but also individually. Here, we are only at the beginning; our study of Turkish/Swedish 4-to-7-year-olds is one such contribution.

Our aim is to present reference data for four TD age groups for a range of tasks that can be utilized in future assessments for DLD. We investigate factors such as age, length of exposure, current daily language exposure and vocabulary size as predictors of performance, and compare the different tasks to each other, to show not only how well the children perform on the tasks themselves, but also to demonstrate how different linguistic skills in the different languages are sensitive to different background variables. Finally, the individual performances of a small sample of DLD children are compared to the TD group, to explore the discriminatory potential of the tasks. This mirrors the situation of speech-language pathologists who are confronted with individual children and not with groups. Our research questions are as follows:

**RQ1:** How do 4–7-year-old Turkish/Swedish-speaking bilinguals with typical language development perform on a language-independent and a language-specific Turkish NWR task, and how is performance affected by age, vocabulary size, and properties of the nonwords (item length and type of task)?
**RQ2:** How do 4–7-year-old Turkish/Swedish-speaking bilinguals with typical language development perform on vocabulary comprehension and production in both languages, and how is that performance affected by age and language exposure?
**RQ3:** How do 4–7-year-old Turkish/Swedish-speaking bilinguals with typical language development perform on comprehension and production of narrative macrostructure in both languages, and how is that performance affected by age, language exposure and vocabulary size?
**RQ4:** By comparison, how do Turkish/Swedish-speaking bilinguals with a diagnosis of DLD perform on these NWR, vocabulary and narrative tasks, and does this allow for any conclusions to be drawn concerning the clinical utility of the LITMUS NWR, CLT and MAIN language tasks when assessing the language skills of bilingual children with suspected DLD?

## 4. Method

### 4.1. Participants

The participants were 114 Turkish–Swedish-speaking children aged 4;0–8;1, all growing up in Sweden.^10^ Of these, 108 had typical language development according to parental report (henceforth, the TD sample), and 6 had a DLD diagnosis from a certified speech-language pathologist, SLP (the DLD sample). The children and their families were part of a larger child multilingualism project, BiLI-TAS (PI: Ute Bohnacker), at Uppsala University.

#### 4.1.1. The TD Sample

The children in the TD sample were recruited by contacting 200 preschools and schools in urban areas of eastern central Sweden. Some children were also recruited via mother-tongue teachers of Turkish, personal contacts of Turkish-speaking members of the research team and associations arranging activities for Turkish-speaking children. One-hundred-and-ten children were recruited for the TD sample, two of whom dropped out of the study as parental consent was withdrawn. One-hundred-and-eight children remained.^11^ Inclusion criteria for participation were (a) being able to speak both Turkish and Swedish (at least to some extent) and (b) having no known hearing problems, language disorders or neuropsychiatric disorders according to parental report. The 108 children in the TD sample attended 50 different preschools and schools. As can be seen in Table 1, the number of participants was fairly even across age groups, with slightly more girls than boys overall.

The vast majority (90%) of the participants were born in Sweden. The remaining 11 children were born in Turkey and had migrated with their families to Sweden. Most children were Turkish/Swedish-speaking bilinguals, but 13 children were exposed via their parents to an additional language other than Turkish and Swedish, and 6 were reported to also speak an additional language (mostly Kurdish).

Age of onset (AoO) for Turkish was at birth for 99 children (the remainder were first exposed to a third language, most often Kurdish, via their parents and exposed to Turkish soon after), and before age 3 for all children (for three children this information was missing). AoO for Swedish was more varied, as only 29 children were exposed to Swedish from birth; however, almost all children started to receive regular exposure to Swedish before age 3 (N = 91). Four children had been exposed to Swedish for less than 24 months. We decided not to exclude children with short residence lengths a priori, as we wanted to explore the effect of length of exposure. Daily exposure (according to parental estimation) varied widely, but was overall a bit higher for Swedish (M = 54.4%, SD = 21.3, Median = 60) than for Turkish (M = 45.1%, SD = 21.1, Median = 40). Younger children (the four-year-olds) tended to have more exposure to Turkish though (M = 49.6%, SD = 20.36, Median = 60).

All children attended institutional childcare. All children at age 4, all but one at age 5, and six children at age 6 were in preschool. In total, 1 five-year-old, 21 of the children at age 6, and 14 of the children at age 7 were in *förskoleklass* (a preparatory year between preschool and primary school), and the remaining 15 7-year-olds were in first grade of primary school. (In Sweden, primary school starts at age 7, but attending state-funded preschools from age 1 or 2 is commonplace.) The children had varying socio-economic backgrounds, with a variety of parental occupations and education levels (all levels from less than six years of primary school to doctorate degrees were represented). The majority of parents had finished secondary school, and about a third had some form of tertiary education. A majority of the children attended (pre)schools in low-SES urban areas with high proportions of multilingual peers and staff.

#### 4.1.2. The DLD Sample

The children in the DLD sample were recruited by contacting SLP clinics and specialized (pre)school units for children with DLD in the same regions as the TD sample. All in all, fourteen children fulfilled the inclusion criteria (see below) and were invited to participate in the study, but only six of them decided to participate in the end. Inclusion criteria were the following: (i) age between 4;0 and 8;1, (ii) being regularly exposed to Turkish and Swedish, and (iii) having a DLD diagnosis. All children had been assessed and diagnosed by a licensed SLP. Diagnoses could include any of the following categories in the ICD-10-SE diagnostic framework, utilized in Swedish healthcare at the time of assessment: mixed receptive and expressive disorder (Swe: *generell språkstörning*), primarily receptive disorder (Swe: *impressiv språkstörning*), primarily expressive disorder (Swe: *expressiv språkstörning*), or grammatical disorder (Swe: *grammatisk språkstörning*), but not exclusively phonological or articulatory difficulties (Swe: *fonologisk språkstörning*). Exclusion criteria were the following: (i) having a biomedical condition associated with language difficulties (e.g., Down syndrome), (ii) a diagnosis within the autism spectrum, or (iii) intellectual disability at the time of assessment. Although not an exclusionary criterion for participating in the DLD study, none of the children had ADHD. Table 2 below and Table A1 in the Appendix A provide more information on the participants’ language impairment.

There were as many girls (3) as boys (3), and the age range (4;9–8;1) matched that of the TD sample.^12^ As can be seen in Table 2, almost all children had mixed receptive and expressive difficulties (‘general language disorder’), but one child had recently had the diagnosis changed to expressive LD, and one child had an unspecified diagnosis at the time of testing since the SLP had not been able to complete the assessment in Turkish, despite several attempts. For another child (BiTurLI-05) it was revealed to the SLP and the research team three months after testing that he had also received an autism diagnosis. However, the parents did not think that this diagnosis was accurate. In the end, it was decided not to exclude this child a posteriori. We decided to do so since the children in the DLD sample are treated as individual case studies rather than as a group. 

Five children had an age of onset of Turkish from birth, and as many had an AoO of Swedish before age 3. BiTurLI-05 was first exposed to Kurdish via his parents, and started to receive regular exposure to Turkish at age 1, and to Swedish at age 2. Three children were reported to have even daily exposure to both languages. One child, BiTurLI-01, had a much higher proportion of Swedish (80%) than Turkish. Two children were exposed to Kurdish in addition to Swedish and Turkish. All children attended institutional childcare. Three children were in preschool, one child attended *förskoleklass*, and one child was in first grade of primary school. The sixth child attended *språkförskola*, a specialized preschool unit for children with severe DLD.

### 4.2. Materials

#### 4.2.1. Nonword Repetition Tasks (NWR)

Two types of NWR tasks were used with the TD and DLD children in the present study, a language-specific one and a quasi-universal/cross-linguistic one. The items in these tasks all adhere to Turkish lexical phonology and syllable structure. Turkish has a rich phoneme inventory, and word-final devoicing, as well as vowel harmony, where vowels agree in backness.^13^ Turkish is an inflectional, agglutinative language, which is syllable-timed (i.e., all syllables, stressed or unstressed, are of roughly equal duration). Turkish syllable structure is mainly CV and CVC, where CV predominates in word-initial syllables, CVC and CV word-medially, and CVC word-finally (Topbaş et al., 2014 [10]). Consonant clusters are uncommon and only occur word-finally. Accordingly, a Turkish language-specific task (LS-Tur) was developed by Seyhun Topbaş, Dilber Kaçar and Handan Kopkallɪ-Yavuz at the Department of Speech and Language Pathology (DİLKOM), Anadolu University, and kindly made available to us. The LS-Tur task contains sixteen test items, with four items of each syllable length (2–5), each item starting with CV and always ending with CVC. Eight of the items are more Turkish-like, as they are made up of high-frequency syllables and have been rated by native-speaker panels as sounding ‘very Turkish-like’ (wordlikeness). The other eight items are less Turkish-like, as they contain syllables that occur at lower frequencies in Turkish and where the item has received lower wordlikeness ratings. All 16 items conform to Turkish phonology (including backness vowel harmony), no phoneme occurs more than once per item, only early-acquired phonemes occur in initial and final position,^14^ and items are made up of open syllables apart from the coda in the final syllable (Topbaş 2015 [75]). An earlier, longer (30-item) version of the LS-task is described in Topbaş et al. (2014 [10]), including the rationale and procedure for item construction, selection and rating, as well as the repetition accuracies of word-like and less word-like items by monolingual Turkish children (aged 4–8 years). The phoneme inventory contains fifteen consonants (/p, t, d, k, ɡ, m, n, s, z, ʃ, d͜ʒ, ʋ, j, l, ɫ/) and nine vowels (/i, y, e, ø, ɛ, a, o, ɯ, u/). The items are pronounced with Turkish prosody, with equal stress on all syllables in terms of duration and pitch, except for final-syllable pitch drop.

Secondly, a cross-linguistic NWR task was used (Chiat 2015 [9], https://www.bi-sli.org/cl-nonword-repetition). Note that this cross-linguistic NWR task was previously called ‘the quasi-universal nonword repetition test’ (Boerma et al., 2015 [16]; Chiat 2015 [9]; Chiat & Polišenská 2016 [49]). This task was used because the ability to repeat quasi-universal items is widely assumed to be transferrable across languages, not to disadvantage bilingual children, and because it has been put forward by several researchers as having the greatest potential to differentiate bilingual TD from bilingual DLD (Section 1). The Turkish version of the task (CL-Tur) was adapted by Topbaş and colleagues at Anadolu University as part of the COST Action IS0804. The CL-Tur task consists of sixteen test items, with four items of each syllable length (2–5) and simple syllabic structure (only open syllables and no clusters). The phoneme inventory is comprised of eleven consonants (/p, b, t, d, k, ɡ, s, z, m, n, l/) and three vowels (/a, i, u/) phonetically realized in Turkish. The items are pronounced with quasi-neutral prosody (equal stress in terms of length and pitch), except for pitch drop of the final syllable, marking the end of an utterance.

Both NWR tasks were professionally recorded in a TV studio by a female native Turkish speaker, cleaned for background noise, and embedded in an audio-visual computer application featuring a parrot with headphones. The items were presented with a three-second pause between each item, starting with the shortest (2 syllables) and ending with the longest items (5 syllables).

In addition to these NWR tasks in Turkish, the DLD children were administered two NWR tasks in Swedish. The Swedish version of the cross-linguistic NWR task (Chiat 2015 [9]), the CL-Swe, is near-identical to the CL-Tur and was developed by the first author for the BiLI-TAS project. It consists of 16 open-syllable items without clusters, 4 nonwords per syllable length (2–5), pronounced with quasi-neutral prosody and pitch drop on the final syllable. The CL-Swe phoneme inventory contains three vowels (/a, i, u/) and ten consonants (/p, b, t, d, k, ɡ, s, m, n, l/), phonetically realized in Swedish. (The CL-Swe inventory thus contains one consonant fewer than the CL-Tur, as /z/ does not exist in Swedish.) The Swedish language-specific NWR task (LS-Swe) is not part of the LITMUS battery, but had previously been developed by two Swedish SLP students (Barthelom & Åkesson 1995 [76]) and was subsequently published (Radeborg et al., 2006 [77]). The LS-Swe contains 24 2-to-5 syllable items that adhere to Swedish phonotactics, with Swedish phonemes, nineteen consonants (/p, b, t, d, k, ɡ, m, n, ŋ, ɾ, f, v, s, ɕ, ɧ, ʂ, h, j, l/) and fifteen vowels (/i, ɪ, y, ʏ, e, ɛ, œ, ɑ, a, o, ɔ, u, ʊ, ʉ, ɵ/). The phonological complexity of the LS-Swe items varies, as they contain open or closed syllables, with and without consonant clusters in onset and coda. Main stress and vowel duration in the nonwords also vary across the syllables, in typical Swedish stress pattern fashion. The LS-Swe and CL-Swe items were prerecorded by a female native speaker of a central Swedish variety.

A list of all the items in the NWR tasks is provided in Table A2 in the Appendix A.

#### 4.2.2. Cross-Linguistic Lexical Tasks (CLTs)

Vocabulary skills were assessed with the Turkish and the Swedish versions of the Cross-linguistic Lexical Tasks (CLT), a picture-based vocabulary assessment material targeting comprehension and production of nouns and verbs (Haman et al., 2015 [30]; Ringblom et al., 2014 [78]; Ünal-Logacev et al., 2013 [79]; https://multilada.pl/en/projects/clt/). The CLT was developed specifically for assessing vocabulary in both languages of bilingual children as part of the COST Action IS0804. Each of the two comprehension parts (nouns, verbs) and each of the two production parts (nouns, verbs) consists of thirty test items preceded by two practice items. Thus, the maximum score is 60 for CLT comprehension and 60 for CLT production. Comprehension is assessed via picture selection. For each test item, the experimenter asks a prompt question (e.g., ‘who is pouring?’) and from an array of four, the child has to select the correct picture. Vocabulary production is assessed via picture naming, where individual pictures are shown to the child one by one. The experimenter asks the child to name a depicted object (e.g., ‘what is this?’) or action (e.g., ‘what is she doing?’).

#### 4.2.3. Multilingual Assessment Instrument for Narratives (MAIN)

Narrative macrostructure comprehension and production was assessed with the Turkish and the Swedish versions of the Multilingual Assessment Instrument for Narratives (MAIN) (Gagarina et al., 2019 [41]) https://main.leibniz-zas.de/en/). The MAIN was developed for assessing narrative macrostructure in both languages of bilingual children as part of the COST Action IS0804. There are four picture sequences in MAIN (Cat, Dog, Baby Birds and Baby Goats), all containing six pictures each. These four stories were designed to be matching in terms of length and story grammar components. Furthermore, two stories each are parallel in terms of plotline and number of characters. Due to this parallelism, we refer to Cat/Dog as one task and Baby Birds/Baby Goats as the other.

#### 4.2.4. Parental Questionnaire

All parents filled in a questionnaire on the social and linguistic background of the participating children and their families. The parental questionnaire was developed as part of a larger research project on child multilingualism (BiLI-TAS) at Uppsala University. The questionnaire could be answered in either Turkish or Swedish, whichever the parents preferred. The questions targeted language exposure, language use in the family, language activities in the home such as book reading and storytelling, language attitudes, parental language skills, and education and occupation, as well as family history of language and/or literacy difficulties, the child’s early language development and parental concerns. The parents of the children in the DLD sample filled in an almost identical questionnaire, with additional questions about SLP referral and therapy.

#### 4.2.5. Interview Data

The interview questions for parents, teachers and SLPs of the DLD children were developed by the BiLI-TAS project research team. Parents were interviewed in connection with the Turkish data collection in the home. The interview targeted the same topics as the questionnaire, but elicited more detailed information about the child’s language development over time and whether the parents were concerned about their child’s language development, as well as the parents’ attitudes and beliefs about bilingualism. Teachers were interviewed in connection with the Swedish data collection at (pre)school. The questions targeted the child’s language and social skills, classroom behavior and learning outcomes. The SLPs were interviewed by telephone after data collection in both languages was done. SLPs were asked about assessment procedures (in which language(s), and which materials were used), language therapy, development over time and the child’s current language status. A condensed overview of the interview data can be found in Table A1 in the Appendix A. For more details on the interviews with parents, teachers and SLPs, see Öztekin (2019 [62]).

### 4.3. Data Collection Procedure

The data were collected between February 2015 and October 2016 for the TD sample, and February through April 2018 for the DLD sample. Families received oral and written information about the project in both Turkish and Swedish. Informed consent was obtained from the parents in writing before data collection, and children gave oral assent. Participation could be discontinued at any time. All children were seen on two separate occasions, one in each language (interval between sessions: M = 13.6, SD = 7.9, range 4–36 days). The meetings took place in a quiet room at (pre)school or in the home. Each session was audio- and video-recorded for later transcription and scoring, and lasted for about 30–45 min. The experimenters were trained native speakers of Turkish and Swedish who spoke to the child only in the language of testing, in order to be able to assess the children’s abilities in each language separately. The order of the languages (Swedish or Turkish first), as well as the order of the tasks, was counterbalanced. The children were assessed with the NWR tasks, the CLT and the MAIN, as part of a test battery that also included a second narrative task (for the TD sample) and two Swedish NWR tasks (for the DLD sample). The child was given stickers and praise at the end of each task.

The NWR tasks were administered via audio-visual computer applications. The tasks were presented to the children as an imitation game, featuring a parrot that the child was instructed to imitate. The task was presented on a laptop and the audio was played to the child via headphones.^15^

The CLT was administered via colored picture booklets and followed the standard procedure described by Haman et al. (2015 [30]). During the session, responses were noted on paper forms. The experimenter gave only neutral feedback (e.g., *aha, mhm, okay*) irrespective of whether the child had provided a correct answer or not. All responses were scored after each session.

The MAIN was administered according to the ‘telling’ mode described by Gagarina et al. (2019 [41]). All children told two stories per language, Cat or Dog and Baby Birds or Baby Goats. First, the child told the story viewing the pictures, but they were not visible to the experimenter. After the child had finished telling, the experimenter laid down the picture sequence on the table for shared visual attention and asked ten standardized questions, all probing inferential comprehension of the macrostructural components of the story.

As a few children did not complete every task in the Turkish and/or the Swedish session, Table 3 provides an overview of how many children per age group, as well as in the sample as a whole, performed which task in the TD sample. In the DLD sample, all children completed all tasks in both languages, apart from BiAraLI-03, who was unable to carry out MAIN BB/BG in Turkish.

### 4.4. Data Treatment

#### 4.4.1. Scoring

All NWR responses from the Turkish session were transcribed and scored by a trained native-Turkish research assistant. The total number of responses was 3488 (1744 for each NWR task: 16 items × 109 participants, 103 TD children and 6 DLD children). A second rater, a native-Turkish PhD student of linguistics and SLP who had also been the experimenter in the Turkish data collection for most of the children, independently scored the NWR responses of a random sample of 14% of the data (12% of the TD children (N = 12), and 50% of the DLD children (N = 3)). Any disagreements were resolved by consensus after discussions with the authors. The authors also checked the scoring of items for internal consistency. Scores (item correct vs. incorrect) matched for 88% (211/240 items) of the checked items for the LS-task, and for 90% (215/240 items) of the items for the CL-task. Inter-rater reliability for item vs. incorrect item (unweighted Cohen’s Kappa) was 0.75 (*p* < 0.001) for the LS-task and 0.74 (*p* < 0.001) for the CL-task. Scoring was carried out as follows. One point was awarded for each correctly repeated item, and 0 points for responses containing an error. Any consistent phonological substitution processes in the child’s speech were disregarded, and allowances were made for indistinct pronunciation of/s/. Errors of voicing (e.g., /p/ vs. /b/) and minor vowel deviations (e.g., /u/ vs. /o/) were also disregarded, but major vowel substitutions (e.g., substituting /a/ for /i/) were not allowed. Following Dollaghan and Campbell (1998 [23]), additions of syllables or phonemes before or after an otherwise correctly repeated item were also disregarded (i.e., children were not penalized for hesitation noises). The responses on the Swedish NWR tasks (administered only to the 6 DLD children), a total of 240 responses ((16 + 24 items) × 6) were transcribed and scored by a trained native-Swedish research assistant and SLP, and checked by the authors. Scoring followed the same principles as the scoring of the Turkish NWR responses.

The CLT forms filled in by the experimenters were checked against the audio and video recordings. There were 25,920 responses (108 participants × 2 languages × 120 test items, 60 for comprehension and 60 for production) in total. Scoring was carried out by trained native or near-native speakers of Turkish and Swedish, respectively. Responses were carefully checked for accuracy and consistency, following project-internal scoring guidelines based on data from 220 monolingual and bilingual children. As there is no standardized published procedure for scoring the CLT, scoring was carried out as follows. One point was awarded for each correct response in the target language, and zero points were awarded for incorrect responses. For the comprehension tasks, only target picture identifications were scored as correct. For the production tasks, a point was awarded for each correctly produced target word. In addition, adult-like synonyms, responses more specific than the target word but still corresponding to the picture, and responses pronounced slightly off-target were also scored as correct. All other responses were scored as incorrect. For more detailed information about transcription and scoring, see Öztekin (2019, pp. 91–92 [62]).

All MAIN narratives, as well as the responses to the comprehension questions, were transcribed orthographically and carefully checked by trained native or near-native speakers of Turkish and Swedish, respectively. Next, all narratives and the responses to the comprehension questions were scored for story structure, according to the standardized procedure described by Gagarina et al. (2019 [41]). The maximum score for story structure production was 17 points, one point for each of the story components that the child mentioned in their narrative: time setting and place setting, plus one point each for internal state as initiating event, goal, attempt, outcome, and internal state as reaction (5 components × 3 episodes). For story comprehension, the maximum score was 10 points. The questions probed the children’s comprehension of the characters’ goals and internal states, as well as inferences about the general plotline. One point was awarded for each of the comprehension questions that were answered correctly. See Gagarina et al. (2019 [41]) and Bohnacker and Gagarina (2020a [69]), for more details.

#### 4.4.2. Statistical Analyses

All statistical analyses were conducted in R (R Core Team 2021 [80]). The level of significance was set at *p* < 0.05 (two-tailed) for all analyses. Correlations were calculated with Pearson’s correlation coefficient (Pearson’s *r*).

Three variables from the questionnaire were investigated with respect to performance on NWR, vocabulary and narrative tasks: chronological age (in months), Length of Exposure to Swedish (LoE Swe) and current daily exposure (daily exp) to Turkish and Swedish. LoE Swe was calculated by subtracting Age of Onset (AoO) for Swedish (in months) from the child’s current age (in months). (As AoO for Turkish was at birth for 92% (99/108) of the children in the TD sample, LoE to Turkish could not be investigated as a separate variable.) The child’s current daily exposure to each language was estimated by the parents on a scale with seven levels ranging from almost only Swedish (95% Swe, 5% Tur) to almost only Turkish (5% Swe, 95% Tur). Parents could also note a different distribution; for example, if the child was exposed to a third language in addition to Turkish and Swedish. Daily exposure was then split into one separate variable for each language: daily exposure to Turkish (daily exp Tur) thus specifies the percentage of daily exposure to Turkish, and daily exposure to Swedish (daily exp Swe) specifies the percentage of daily exposure to Swedish.

Accuracy was investigated for the Turkish NWR tasks with a logistic mixed-effects regression model, using the function *glmer* from the *lme4* package (Bates et al., 2015 [81]). The mixed-effects model was evaluated with pseudo-R^2^ and concordance index (c-index). Pseudo-R^2^ was obtained with the *r.squaredGLMM* function from the MuMIn package (Bartoń 2020 [82]). See Section 5.1 for more information about the model.

For vocabulary and narrative macrostructure, multivariate linear regression models were fitted with the vocabulary or narrative macrostructure score as the dependent variable. Comprehension and production was investigated separately for each language, thus there were eight separate models. For more information about specific models, see Section 5.1, Section 5.2 and Section 5.3.

Finally, age-adjusted z-scores were calculated, in order to compare individual performances of the DLD children on the NWR, vocabulary and narrative macrostructure tasks to those of the children in the TD sample. All individual z-scores were calculated based on the mean and SD for the children in the corresponding age group in the TD sample. Thus, all z-scores indicate how each individual performed on a specific task compared to age-group peers in the TD sample.

## 5. Results: The TD Sample

### 5.1. NWR

First, scores are reported by age group for the two NWR tasks. As evident in Table 4, overall performance was slightly lower in the LS-Tur task than in the CL-Tur task for all age groups, and mean scores increased with age among all age groups. In the following, age will be treated as a continuous variable. There were positive correlations between linear age and scores on both NWR tasks (LS-Tur: *df* = 101, *r* = 0.28, *p* = 0.004; CL-Tur: *df* = 101, *r* = 0.29, *p* = 0.003). However, scores did not increase substantially with age, as evident from the small effect sizes of the correlations, and only small differences in mean scores between the age groups (see Table 4). Note that this was not due to a ceiling effect, since even the mean scores for the seven-year-olds were several points below the maximum score. Only for the LS-Tur task was there a (positive) correlation with Turkish vocabulary (comprehension: *df* = 96, *r* = 0.21, *p* = 0.04), production: *df* = 96, *r* = 0.22, *p* = 0.03) but not for the CL-Tur task.^16^

**Figure 1:**
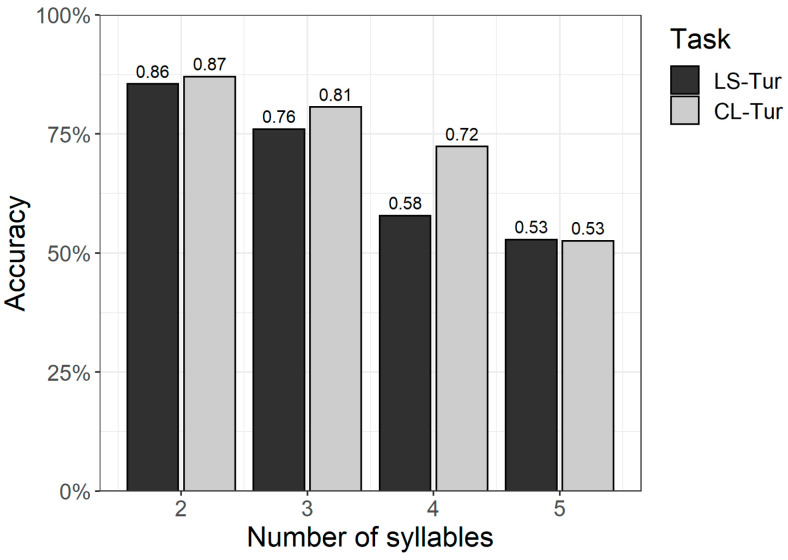
Accuracy (% correct responses) for the LS-Tur and the CL-Tur task by number of syllables.

Next, accuracy (percent correctly repeated items) was investigated in relation to item length (number of syllables). As shown in Figure 1, accuracy is the highest for the shortest items (2 syllables) in both tasks, and gradually decreases for each added syllable. For three- and four-syllable items, accuracy is somewhat higher on the CL-Tur task. For the shortest as well as the longest items (2 and 5 syllables, respectively), accuracy is similar in both tasks.

A logistic mixed-effects regression model (Model 1, see Table A3 in the Appendix A for a summary) was fitted in order to investigate the effect of item-related factors and participant-related factors on repetition accuracy. The model investigated the effect of age, Turkish vocabulary comprehension,^17^ task (CL-Tur vs. LS-Tur), item length (number of syllables), and the interaction between task and Turkish vocabulary on repetition accuracy in both NWR tasks (fixed effects). By-participant and by-item intercepts were included in the model as random effects. While chronological age (B = 0.20, SE = 0.09, *p* = 0.02) had a positive effect on repetition accuracy, an increasing number of syllables had a negative impact (B = −0.82, SE = 0.16, *p* < 0.001). While there were no main effects of Turkish vocabulary scores (B = −0.05, SE = 0.10, *p* = 0.64) or task (B = −0.42, SE = 0.31, *p* = 0.17), there was a significant interaction between task and vocabulary (B = 0.21, SE = 0.09, *p* = 0.02), demonstrating that vocabulary size positively affected repetition accuracy for LS-Tur items but not for CL-Tur items. The explanatory power of the full model was considerable (conditional R^2^ = 0.37) and better than that of the fixed effects alone (marginal R^2^ = 0.15). The C-index of 0.82 indicates a good model fit.

### 5.2. Vocabulary

First, we present CLT scores in the two languages separately for each age group. As Table 5 shows, mean scores increased with age for comprehension and production in both languages. Scores are presented here for age groups so that they are able to be used as reference data, but in the following, age will be treated as a continuous variable. Linear age correlated positively with Turkish comprehension (*df* = 100, *r* = 0.28, *p* = 0.005), Swedish comprehension (*df* = 100, *r* = 0.62, *p* < 0.001) and Swedish production (*df* = 100, *r* = 0.61, *p* < 0.001). For Turkish production, the trend was similar, but failed to reach significance (*df* = 100, *r* = 0.19, *p* = 0.06). (This was likely due to substantial individual variation depending on factors other than age).

Four multivariate linear regression models were run to investigate the joint effect of age, estimated daily exposure and length of exposure (for Swedish) on Swedish and Turkish vocabulary scores. Summaries of the regression models can be found in Table A4 and Table A5 in the Appendix A.

For Turkish comprehension scores, age, length of exposure to Swedish and daily exposure to Turkish explained 20% of the variance (Model 2). Standardized estimates showed that age (β = 0.50, *p* < 0.001) was a stronger predictor than daily exposure to Turkish (β = 0.25, *p* = 0.009) for Turkish comprehension scores. There was also a tendency for length of exposure to Swedish to be a negative predictor of Turkish comprehension scores (β = −0.22, *p* = 0.057). For Turkish production, age, length of exposure to Swedish and daily exposure to Turkish explained 19% of the variance in scores (Model 3). Just like for comprehension, age was the strongest predictor (β = 0.44, *p* < 0.001), followed by a negative effect of length of exposure to Swedish (β = −0.33, *p* = 0.005) and finally a positive effect of daily exposure to Turkish (β = 0.26, *p* = 0.005).

As for Swedish vocabulary, age, length of exposure to Swedish and daily exposure explained 49% (Model 4) of the variance in comprehension scores, with age (β = 0.38, *p* < 0.001) being a slightly stronger predictor than length of exposure (β = 0.34, *p* < 0.001) and daily exposure (β = 0.16, *p* = 0.03). For Swedish production, age, length of exposure and daily exposure explained 53% of the variance (Model 5). Age (β = 0.36, *p* < 0.001) and length of exposure (β = 0.34, *p* < 0.001) were stronger predictors than daily exposure (β = 0.26, *p* < 0.001). Thus, the models investigating Swedish vocabulary explained more than twice as much of the variance in scores compared to the models investigating Turkish vocabulary.

### 5.3. Narrative Macrostructure

First, we present the MAIN scores separately for each age group (Table 6). There were four scores per child, one for macrostructure comprehension and one for macrostructure production in Turkish and Swedish, respectively. For simplicity, the story structure scores for MAIN Cat/Dog and Baby Birds/Baby Goats have been combined here. (Descriptive statistics for the two tasks separately (Cat/Dog, Baby Birds/Baby Goats) are found in Table A8 and Table A9 in the Appendix A) Table 6 shows that the mean scores for comprehension and production increase with age in both languages. Scores for age groups are provided here as reference data, but in the following, age will be treated as a continuous variable. Linear age correlated positively with Turkish comprehension (*df* = 100, *r* = 0.43, *p* < 0.001) and production (*df* = 100, *r* = 0.55, *p* < 0.001), as well as with Swedish comprehension (*df* = 100, *r* = 0.68, *p* < 0.001) and production (*df* = 100, *r* = 0.66, *p* < 0.001).

Four multivariate regression models were run to investigate the joint effect of age and vocabulary on narrative scores, controlling for language exposure in terms of length of exposure to Swedish and percent daily exposure to Turkish/Swedish. The regression models are summarized in Table A6 and Table A7 in the Appendix A.

Model 6 explained 53% of the variance in Turkish macrostructure comprehension scores. Standardized estimates showed that Turkish vocabulary production was the strongest predictor (β = 0.62, *p* < 0.001), followed by age (β = 0.27, *p* = 0.004). Length of exposure to Swedish (β = 0.06, *p* = 0.55) and daily exposure to Turkish (β = 0.02, *p* = 0.82) were not significant predictors.^18^ For Turkish macrostructure production, Model 7 explained 57% of the variance. Again, Turkish vocabulary production (β = 0.56, *p* < 0.001) was a stronger predictor than age (β = 0.36, *p* < 0.001). Just like for comprehension, neither length of exposure to Swedish (β = 0.12, *p* = 0.17) nor daily exposure to Turkish (β = 0.04, *p* = 0.61) were significant predictors of Turkish narrative production scores.

For Swedish macrostructure comprehension scores, Model 8 explained 64% of the variance. Swedish vocabulary production was the strongest predictor (β = 0.60, *p* < 0.001), followed by age (β = 0.36, *p* < 0.001). Neither of the exposure measures were significant (length of exposure to Swedish: β = −0.06, *p* = 0.51, daily exposure to Swedish: β = −0.06, *p* = 0.36).^19^ Model 9 explained 61% of Swedish macrostructure production scores, with Swedish vocabulary production being a stronger predictor (β = 0.55, *p* < 0.001) than age (β = 0.32, *p* < 0.001). Again, neither length of exposure to Swedish (β = 0.00, *p* = 0.99) nor daily exposure to Swedish (β = 0.00, *p* = 0.96) were significant predictors.

### 5.4. Summary: NWR, Vocabulary, and Narrative Macrostructure in the TD Sample

Summarizing the results for the TD sample, for NWR, there was only a little development with age. Performance was slightly higher on the cross-linguistic task (CL-Tur) than on the language-specific task (LS-Tur). As item length increased, repetition accuracy decreased with each added syllable for both tasks. When age was accounted for in the multivariate model, there was a positive effect of Turkish vocabulary scores only for the LS-Tur task.

For vocabulary, development with age was found in both languages and both modalities, though it was more pronounced for Swedish than for Turkish. Age, length of exposure to Swedish and percentage daily exposure (to the specific language) explained more of the variation in Swedish vocabulary scores than for Turkish. Age was the most important predictor of vocabulary scores in both languages and modalities, overshadowing exposure measures. Percent daily exposure to Turkish and Swedish in the respective language was a positive predictor of vocabulary scores in both Turkish and Swedish. Length of exposure to Swedish was a positive predictor of Swedish vocabulary comprehension and production scores, and a negative predictor of Turkish production scores.

For narrative macrostructure, we found a development with age in both languages and both modalities. Vocabulary production scores in the respective language were the strongest predictors of macrostructure scores for comprehension and production in both languages, followed by chronological age. Interestingly, language exposure measures (length of exposure to Swedish and daily exposure to the corresponding language) were not significant predictors of narrative macrostructure in either language or modality.

## 6. Results: The DLD Sample

### 6.1. Profiles of the Individual Children

This section compares the performance of the DLD children to their TD peers. The comparison is made by transforming each child’s raw scores into an age-adjusted z-score (based on the mean and SD for that task for the corresponding age group in the TD sample). We opted here for a z-score below −1.25 as a cut-off, which identifies the lowest-scoring 10.6% of an age group on a specific task (Tomblin et al., 1997 [83]).

**Figure 2:**
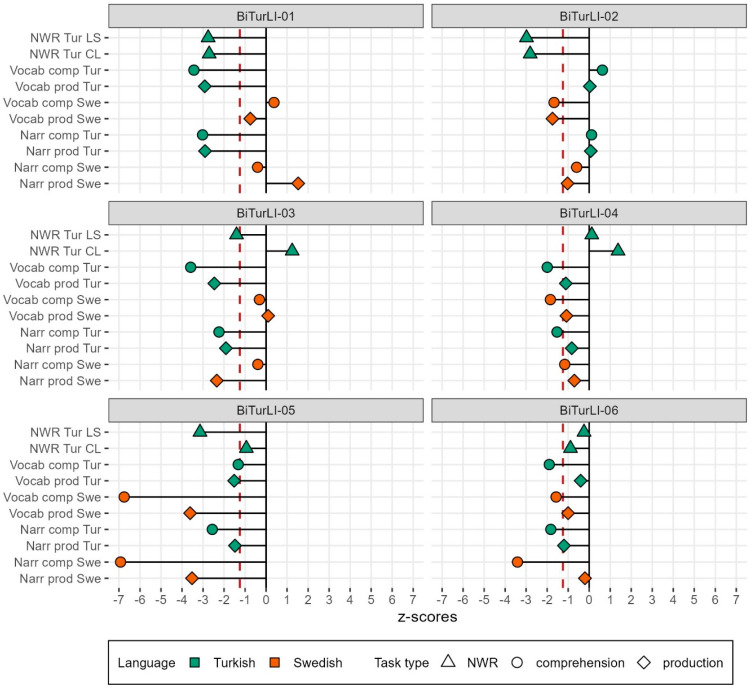
Age-adjusted z-scores for all tasks for each participant in the clinical sample. Black solid line at 0; red dashed line at −1.25.

Individual scores for all tasks are presented in Table 7 (raw scores and z-scores) and Figure 2 (z-scores). Performance was mostly below the mean, as evident by the predominantly negative z-scores. Looking at individual profiles, some children had a clear discrepancy in performance between the two languages; BiTurLI-01, for instance, had very low scores in Turkish and within the typical range in Swedish, while BiTurLI-02 had low scores in Swedish but average scores in Turkish. Interestingly, vocabulary z-scores of the DLD children were generally lower for comprehension than for production, particularly in the minority language (Turkish). A similar pattern emerged for narrative macrostructure, with overall lower z-scores for comprehension in Turkish. Furthermore, the five children who had a z-score below −1.25 in Turkish vocabulary comprehension all scored below −1.25 in Turkish narrative macrostructure comprehension. One child (BiAraLI-05) had very low scores across the board, particularly in Turkish and Swedish vocabulary and narrative comprehension.

### 6.2. NWR

**Figure 3:**
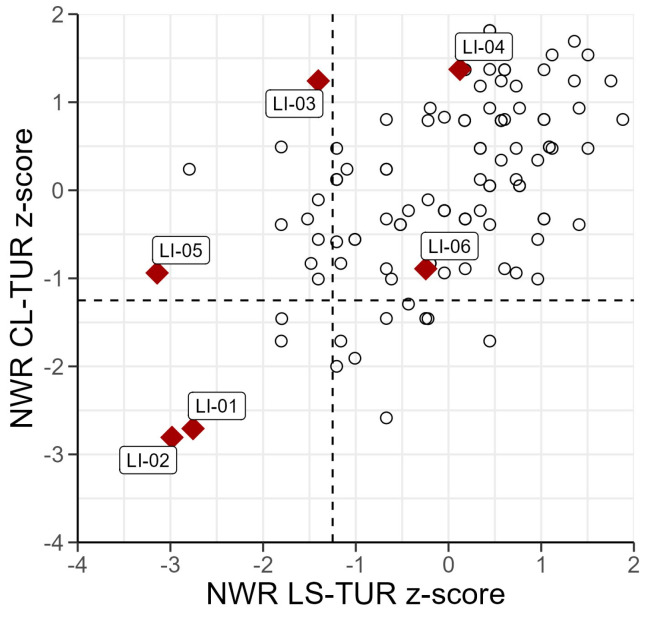
Age-adjusted z-scores of the LS-Tur and the CL-Tur NWR task for the children in the DLD sample (diamonds and labels) compared to the children in the TD sample (circles). Dashed lines at −1.25.

Figure 3 shows age-adjusted z-scores plotted for the two NWR tasks for the children in the TD and the DLD samples. Four children in the DLD sample scored below the −1.25 cut-off on the LS-Tur, but only two children scored below the cut-off on the CL-Tur. Notably, the DLD children were scattered all over the plot, with two children scoring well below the cut-off in both tasks, and one child (BiTurLI-04) scored remarkably well in both tasks.^20^ Overall, there was a substantial overlap in performance between the TD and the DLD groups.

There were three children in the TD sample who performed below −1.25 in both tasks. Two of them had normal language development according to parental report and the parents did not express concern about their children’s language. When inspecting the video recordings from the Turkish session it became evident that these children had trouble understanding the task and therefore performed poorly. The third child was reported to have late language development, and the parents voiced concern about the language abilities of their child.

In addition to the two NWR tasks in the Turkish session, the DLD children also did two NWR tasks in the Swedish session: the Swedish version of the cross-linguistic task (CL-Swe) and a language-specific Swedish task (LS-Swe). A table with individual raw scores and proportional scores (%) is available in Table A10 in the Appendix A. As evident from Table A10, performance on the two cross-linguistic tasks was similar across language versions (three children’s scores differed by one point, two by two points, and one by three points). The similar performance on both tasks was confirmed by a correlation analysis (*df* = 4, *r* = 0.95, *p* = 0.003). Compared to the cross-linguistic NWR tasks, all children scored proportionally lower (or, at best, the same) on the two language-specific NWR tasks.

### 6.3. Vocabulary

**Figure 4:**
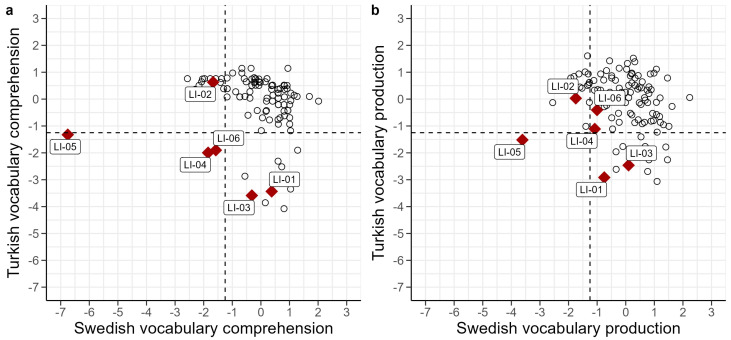
Age-adjusted z-scores of (**a**) Turkish and Swedish vocabulary comprehension and (**b**) Turkish and Swedish vocabulary production for the children in the DLD sample (diamonds and labels) compared to the children in the TD sample (circles). Dashed lines at −1.25.

In Figure 4, age-adjusted z-scores are plotted for Turkish and Swedish vocabulary comprehension (a) and production (b) for the TD and the DLD children. All DLD children scored below the −1.25 cut-off for comprehension in at least one of the languages, but only three scored below in both languages. For production, four children scored below the cut-off in one language, but only one in both languages. Two children in the DLD sample also scored above the cut-off in both languages. The DLD and TD samples showed some overlap, and this overlap was more pronounced for production than for comprehension. Most TD children are in the upper right quadrant with scores above the −1.25 cut-off in both languages. The ones performing below the cut-off in one language tend to perform above or just below the mean in the other language, i.e., they have uneven proficiency levels.

Next, we wanted to explore whether these uneven vocabulary scores were related to language exposure. LoE Swe (in months) was transformed into age-adjusted z-scores in order to be comparable across the age groups. The TD children that fell below the cut-off in Turkish (N_comp_ = 7, N_prod_ = 10) had generally been exposed to Swedish longer (age-adjusted z-scores: M_comp_ = 0.4, SD = 0.9, M_prod_ = 0.4, SD = 0.8) and had less daily exposure to Turkish (percent exposure: M_comp_ = 30%, SD = 10%, M_prod_ = 30%, SD = 10%) compared to the whole-group average. By contrast, the TD children that fell below the cut-off in Swedish (N_comp_ = 13, N_prod_ = 17) had generally been exposed to Swedish for a shorter time (age-adjusted z-scores: M_comp_ = −1.0, SD = 1.2, M_prod_ = −1.0, SD = 1.0) and had less daily exposure to Swedish (percent exposure: M_comp_ = 50%, SD = 20%, M_prod_ = 40%, SD = 20%) compared to the whole-group average. This finding aligns with the results of Models 2–5, demonstrating an association between language exposure and vocabulary scores.

We then reviewed the exposure measures for the children in the DLD sample with uneven vocabulary scores in order to explore whether exposure patterns could provide an explanation for their results. BiTurLI-01 scored well below the cut-off in both Turkish comprehension and production, but within 1 SD from the mean in Swedish comprehension and production. BiTurLI-01 had 80% daily input in Swedish, which could provide an explanation for the proportionally higher Swedish scores. BiTurLI-02 was relatively stronger in Turkish than Swedish. He had a higher proportion of Turkish (60%) than Swedish (40%) in his daily input, but his Swedish was unexpectedly low considering that onset of exposure to Swedish was at age two. Similarly to BiTurLI-01, BiTurLI-03 scored close to the mean in Swedish, but his Turkish scores fell well below average, despite an age of onset for Turkish at birth and receiving continuous daily exposure to Turkish (50%). BiTurLI-05 scored below average in both Turkish and Swedish, but his Swedish vocabulary scores were much lower than his Turkish vocabulary scores, despite equal daily exposure to Turkish (40%) and Swedish (40%) (the remaining 20% exposure was to Kurdish, recall Table 2). In summary, exposure measures seemingly provide an explanation for uneven vocabulary scores in one of the DLD children, but, otherwise, vocabulary scores were not generally concurrent with exposure measures.

### 6.4. Narrative Macrostructure

**Figure 5:**
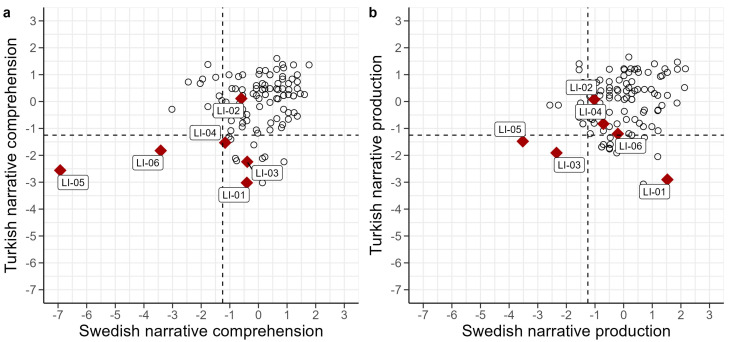
Age-adjusted z-scores of (**a**) Turkish and Swedish narrative macrostructure comprehension and (**b**) Turkish and Swedish narrative macrostructure production (MAIN story structure) for the children in the DLD sample (diamonds and labels) compared to the children in the TD sample (circles). Dashed lines at −1.25.

Figure 5 plots age-adjusted z-scores for Turkish and Swedish narrative macrostructure comprehension (a) and production (b) for the TD and the DLD children. In comprehension, five of the DLD children scored below the −1.25 cut-off in one language (Turkish), but only two did so in both languages. In production, three children performed below the cut-off in one language (again, Turkish), but only two children scored below in both languages. Just like for vocabulary, there was more overlap between the TD and DLD samples in production than in comprehension. Most TD children scored above the cut-off in both languages. There were also a few TD children who fell below the cut-off in one of the languages; most often, they performed above or close to the mean in the other language.

In order to explore the effect of vocabulary production on narrative macrostructure for these low-scoring TD children, vocabulary scores were transformed into age-adjusted z-scores in order to be comparable across the age groups. The TD children that fell below the cut-off in Turkish narrative macrostructure (N_comp_ = 10, N_prod_ = 10) generally had lower Turkish vocabulary production scores (age-adjusted z-scores: M_comp_ = −1.5, SD = 1.3, M_prod_ = −1.3, SD = 1.5) compared to the whole-group average.^21^ Likewise, the TD children that fell below the cut-off in Swedish (N_comp_ = 12, N_prod_ = 10) generally had lower Swedish vocabulary production scores (age-adjusted z-scores: M_comp_ = −1.1, SD = 1.1, M_prod_ = −1.3, SD = 1.0) compared to the whole-group average.^22^ Thus, scoring below the −1.25 cut-off in narrative macrostructure was associated with low expressive vocabulary skills in that language. This finding aligns with the results of Models 6–9, demonstrating an association between vocabulary production scores and narrative macrostructure scores.

Also, for the DLD children, low vocabulary scores were typically associated with low narrative macrostructure scores in the corresponding language (Turkish or Swedish) and modality (comprehension or production).

### 6.5. NWR, Vocabulary, and Narrative Macrostructure in the DLD Sample: Summary

In summary, the children in the DLD sample scored lower than the children in the TD sample on all tasks. There were, however, individual differences which manifested in different profiles, with individual strengths or weaknesses in one of the languages. For NWR, there was a considerable overlap in performance between the TD and the DLD group, which is notable as NWR is frequently put forward as a clinical marker for DLD and one that also works well for bilingual children. Although the TD and the DLD groups also showed some overlap in vocabulary and narrative macrostructure, there was a clearer difference in performance for Turkish vocabulary comprehension and narrative comprehension than for production, with the DLD children generally performing below the −1.25 z-score cut-off on both comprehension tasks.

## 7. Discussion

### 7.1. Factors Affecting Performance on NWR, Vocabulary, and Narrative Macrostructure in the TD Sample

We found that performance increased with age for both NWR tasks, the LS-Tur (Topbaş et al., 2014 [10]) and the CL-Tur (Chiat 2015 [9]), and that these age effects held also when controlling for vocabulary scores. This is consistent with several previous studies showing development with age for NWR tasks in other populations (Kalnak et al., 2014 [11]; Öberg & Bohnacker 2022 [14]; Topbaş et al., 2014 [10]). For both the LS-Tur and the CL-Tur, accuracy decreased as item length (i.e., number of syllables) increased, in line with previous studies using other language versions of the cross-linguistic NWR task, as well as studies using different stimulus items (Boerma et al., 2015 [16]; Öberg & Bohnacker 2022 [14]; Schwob et al., 2021 [13]; Topbaş et al., 2014 [10]). Higher vocabulary scores were associated with better NWR performance, but this effect held only for the LS-Tur task when controlling for chronological age. This finding supports evidence from previous studies demonstrating an effect of vocabulary on language-specific NWR tasks (Öberg & Bohnacker 2022 [14]; Sorenson Duncan & Paradis 2016 [19]; Thordardottir & Brandeker 2013 [21]).

As regards vocabulary, chronological age, length of exposure to Swedish and percent daily exposure (to Turkish/Swedish) explained more than twice as much of the variance in the children’s Swedish comprehension and production scores compared to their Turkish scores. For Turkish comprehension, this result might be explained by a tendency for a ceiling effect, as scores were already high overall among the four-year-olds (M = 51.4, i.e., 86% correct), and individual variation was smaller compared to Turkish production and Swedish comprehension and production scores (recall Table 5). By contrast, for Turkish production, there was no ceiling effect and large individual variations in scores. Thus, for vocabulary in the minority language, particularly expressive vocabulary, crude measures of exposure length or percent daily exposure could not sufficiently explain vocabulary growth. LoE to Swedish added unique explanatory value to both Swedish comprehension and production scores, with an effect size as large as that of chronological age and larger than that of percent daily exposure to Swedish. Interestingly, LoE to the majority language (Swedish) was also a significant negative predictor for minority language (Turkish) production scores, with a somewhat larger effect size than percent daily exposure to Turkish.

For both narrative macrostructure comprehension and story structure production in Turkish and Swedish, we found a clear development with age. Interestingly, exposure measures were not significant predictors for macrostructure in either language or modality; rather, vocabulary production scores emerged as the most important predictor of narrative macrostructure scores, overshadowing chronological age. Thus, expressive narrative macrostructure skills in both Turkish and Swedish seem to be affected by the same factors (namely, vocabulary size and age, but not quantitative measures of language exposure). Additionally, we found that these patterns were also alike for *comprehension* of narrative macrostructure in the majority language, Swedish (the language of schooling and society) and in the minority language, Turkish (the language of the home). We interpret the finding that both age and vocabulary were strong, uniquely contributing predictors for all macrostructure measures, as follows. Older children are cognitively more mature and likely to have more experience with stories, which makes it easier for them to interpret causal, hierarchical and thematic relations between depicted story events, and relate them to a listener. Yet, for children with only limited lexical skills in the language in question, it is difficult to verbally communicate the plot and point of the story and its macrostructural components to a listener, both when telling the story and when answering comprehension questions (see also, Bohnacker et al., 2020 [84]; 2022 [85]). The proposal that narrative macrostructure skills are easily transferred from the L1 to the L2 and quickly put to use there (Iluz-Cohen & Walters 2012 [65]; Paradis et al., 2021, pp. 170–171 [18]; Pearson 2002 [40]) would thus have to be qualified and made contingent on sufficient expressive vocabulary in the L2.

The results of this study indicate that depending on the task at hand, different aspects of a bilingual child’s linguistic background and their proficiency in other language domains need to be taken into account when interpreting their performance on language tests. For instance, TD children with low vocabulary scores may be disadvantaged compared to their peers who have high vocabulary scores on language-specific NWR tasks and narrative macrostructure comprehension and production tasks. Vocabulary scores, unlike narrative macrostructure scores, are strongly affected by quantitative measures of exposure (LoE to the majority language and proportion of daily exposure to the language of testing), and in different ways, depending on language status (minority vs. majority) and modality (comprehension vs. production). For example, TD children with longer LoE to the majority language may be clearly disadvantaged (compared to their peers with shorter LoE) on minority-language vocabulary production tasks; however, they may not be at a clear disadvantage for minority-language vocabulary comprehension. These insights have implications for the linguistic assessment of bilinguals with suspected DLD.

### 7.2. NWR, Vocabulary, and Narrative Macrostructure in the DLD Sample

In this section, we discuss the performance of the DLD children on the NWR, vocabulary and narrative tasks.

Concerning *nonword repetition*, there was substantial individual variation, with a lot of overlap between the two groups (TD and DLD), for both the language-independent and the Turkish language-specific NWR task. Only two children in the DLD sample scored below the −1.25 z-score cut-off, and one DLD child scored well below the cut-off in one task, but just above in the other. An additional two DLD children had a large discrepancy (high scores on one NWR task, but low scores on the other task), and one child scored high in both tasks. Neither the CL-Tur (Chiat 2015 [9]) nor the LS-Tur (Topbaş et al., 2014 [10]) were useful for identifying DLD in our sample of Turkish–Swedish-speaking bilinguals. A similar overlap in NWR performance of TD and DLD children has been found for 109 Arabic–Swedish-speaking bilinguals of the same age as the present sample (Öberg & Bohnacker 2022 [14]). 

Unlike Boerma et al. (2015 [16]) we did not find the CL-NWR to be a better diagnostic tool than the LS-task. There could be multiple reasons for these divergent findings. One explanation might lie in differences in the lexical phonology of Turkish vs. Dutch, with Turkish syllable structure being less phonologically complex and thus easier to repeat for both DLD and TD children. Another explanation could be that the bilinguals in our study were presented with a language-specific NWR task in their L1 Turkish whereas Boerma et al. (2015 [16]) used a language-specific NWR task in the children’s L2 Dutch. (However, remember that our DLD children were tested on language-specific NWR tasks in both Turkish and Swedish in addition to the Turkish and Swedish versions of the cross-linguistic NWR tasks. Performance was generally lower on both language-specific tasks, even though the Turkish language-specific task is very dissimilar from the LS-Swe task (which includes many clusters and a variety of open and closed syllables) and more similar to the cross-linguistic tasks with regard to phonological setup (due to its lack of consonant clusters and predominance of open syllables.)) A third explanation that we find plausible is that of differences in sampling. Boerma et al. (2015 [16]) only assessed the children in their L2 Dutch, and their two groups were heavily preselected. An inclusion criterion for their DLD group was scoring below −2 SD (composite score), or −1.5 SD on two subscales on a standardized test battery. As Boerma et al. acknowledge, their research design may have boosted diagnostic accuracy, as NWR is known to classify TD vs. DLD less well in population-based samples compared to preselected groups (Ellis Weismer et al., 2000 [86]). Our findings are consistent with those of Polišenská et al. (2022 [59]), who reported that the CL-NWR could not reliably discriminate bilingual TD children from their peers with DLD, in a large-scale study combining datasets from 18 research teams in 15 countries. Our results also align with multiple reports of a higher degree of TD/DLD overlap and poorer NWR diagnostic accuracy for identifying DLD in bilinguals (dos Santos & Ferré 2018 [53]; Schwob et al., 2021 [13]; Thordardottir & Brandeker 2013 [21]). Taken together, our discouraging results regarding the use of NWR to identify DLD in bilinguals can be understood in light of two recent meta-analyses that report evidence for a publication bias for studies finding positive and strong effects of NWR as a clinical marker for DLD in bilinguals (Ortiz 2021 [12]; Schwob et al., 2021 [13]).

Moving on to the *vocabulary* tasks, all DLD children scored below the cut-off for vocabulary comprehension in at least one of their languages, most often in Turkish, but only three did so in both languages. For vocabulary production, four children scored below the cut-off in one language, but only one in both languages. There was some overlap in individual scores between the TD and the DLD groups, but more so for production than for comprehension. These results do not match those of Khoury Aouad Saliby et al. (2017 [61]) who found less overlap in production scores between the TD and the DLD group in their sample of Arabic/French/English-speaking bilinguals (although they used conceptual scoring and did not factor in age in their analyses). The scores of the TD and the DLD children in the present study overlapped less than those of the TD and DLD Arabic–Swedish-speaking bilinguals of the same age in Öberg & Bohnacker (2022 [14]). Note, however, that the DLD sample in our study was only half as large (N = 6) as that of Öberg and Bohnacker (N = 11). We did not find any evidence for Haman et al.’s (2015, p. 198 [30]; Haman & Łuniewska 2013 [31]) contention that lexical deficits in *both* languages are a warning sign for DLD.

For *narrative macrostructure*, five of our six DLD children scored below the cut-off in comprehension in the minority language, Turkish. As mentioned in Section 2.3, there are very few studies comparing MAIN narrative macrostructure in bilingual TD vs. DLD children, and they have only investigated comprehension and production in the L2. Similarly to Boerma et al. (2016 [72]) and Boerma and Blom (2017 [22], 2021 [17]), we found that narrative comprehension differentiated the TD and DLD children better than narrative production. Gagarina et al. (2015, p. 264 [87]) suggested that MAIN can be used for screening and identification of bilinguals at risk of DLD. In our study, which included both languages, narrative comprehension in the minority language (Turkish) was more differentiating than minority language production or majority-language narrative measures.

Interestingly, nonword repetition and narrative skills, the two types of tasks that have widely been proposed as suitable to use in bilingual assessment due to their more general-cognitive and less exposure-dependent nature (e.g., Boerma et al., 2015 [16]; Boerma & Blom 2021 [17]; Paradis et al., 2013 [33], 2021, pp. 170–171 [18]; Thordardottir & Brandeker 2013 [21]), were not the most differentiating tasks in our study. Rather, out of all measures included, CLT vocabulary comprehension scores (particularly in the minority language) seemed to best identify the bilingual children with DLD. The finding that the language comprehension scores better identified the DLD children in our sample does not mean that their problems with comprehension are greater than with production. Rather, the TD children cluster together more tightly for comprehension (and are scattered more for production), which makes low comprehension scores stand out more for the DLD children. Of course, it is difficult to make generalizations from these findings, since our sample of DLD children is small and DLD is a heterogeneous disorder. Still, our findings align with those of Smolander et al. (2021 [60]), who found (although they only investigated the majority language) that vocabulary tests were informative in the assessment and diagnosis of DLD in their sample of Finnish bilinguals, when exposure history was taken into account.

It is noteworthy that the DLD children in the present study, *despite* extensive and sustained exposure to the minority language, Turkish, had very low vocabulary-comprehension scores in that language, and a majority of them also had very low vocabulary comprehension in the majority language, Swedish, *despite* extensive exposure to Swedish. By contrast, the TD children scored high on vocabulary comprehension when extensively exposed to the language in question. This could suggest that bilingual DLD children make less efficient use of the language input they receive (Govindarajan & Paradis 2019 [32]).

### 7.3. Individual Children in the DLD Sample: Language Scores in Relation to Exposure and Functional Communication

As seen in Section 6.1, there was great variation in the language profiles in the DLD group. Some variation with regards to language proficiency and communication difficulties in the DLD group can be attributed to the heterogeneity of the DLD diagnosis, which means that a child may have individual strengths and weaknesses in one modality (i.e., comprehension or production) and/or in a particular linguistic domain. Another source of variation is language exposure history. In this section, the language scores of the DLD children will be discussed in light of the information obtained from the parent, teacher and SLP interviews, as well as the experimenters’ observations during assessment (summarized in Table A1 in the Appendix A). While exposure seemingly provided a partial explanation for the performance of one child, it did not provide an explanation for the poor performance in all children.

BiTurLI-01 (age 6;3) was one of only two DLD children whose scores fell well below the cut-off in both NWR tasks. Her proficiency in Turkish and Swedish was uneven, as reflected in much higher z-scores in Swedish vocabulary and narrative macrostructure, compared to the scores in Turkish. Regular exposure to Swedish started early (between the age of one and two), and daily exposure was mostly in Swedish (80%). This child’s Swedish vocabulary and narrative scores were within 1 SD from the TD mean for her age, and for narrative production even above 1 SD from the mean, but several SDs below the mean in Turkish. If we were to consider only these language scores and exposure measures, we could be led to believe that this was simply a TD Swedish-dominant child as a result of uneven exposure. However, BiTurLI-01 attended a Swedish-medium *språkförskola* due to severe DLD, where she received a lot of tailored support to develop her Swedish language and communication skills on a daily basis. According to parental report, neither her Turkish nor Swedish were strong, and communicative problems were frequent. The teacher described frequent misunderstandings and peer conflicts in preschool, and the SLP pointed out that progress in therapy was very slow. Thus, the child’s relatively high Swedish vocabulary and narrative scores on the LITMUS tasks did not match the *described* and *observed* language and overall communication skills. Here, problems were reported, despite the fact that BiTurLI-01 had an early onset and a large proportion of daily exposure to Swedish and received a lot of language and communication support. The child’s middling performance on the Swedish vocabulary and narrative tasks may thus be partly due to an effect of intensive therapy in Swedish.

BiTurLI-02 (age 5;4) was the only DLD child who performed relatively well in Turkish, as all Turkish vocabulary and narrative tasks were close to the age-group mean score. His Swedish was comparatively weaker, as both vocabulary and narrative macrostructure scores were well below the age-group mean. Exposure to Swedish had started between age two and three, and daily exposure to Turkish was relatively high (60%). In this case, it was unsurprising that his Turkish scores were high, but his Swedish scores were unexpectedly low, considering that he had regular exposure to Swedish for about three years and attended a Swedish-medium preschool. Interviews with parents, teachers, and the child’s SLP were consistent with his language scores, as BiTurLI-02 was consistently described to have very poor Swedish, and to speak Turkish to interlocutors who did not understand any Turkish. At preschool he constantly sought help from other Turkish-speaking peers, and the preschool teacher reported that the child had great difficulties remembering words in Swedish. The SLP reported that his Swedish was virtually ‘non-existent’ and that he had great difficulties learning new words in Swedish. Despite having age-appropriate scores in Turkish, BiTurLI-02 had great difficulties with functional communication, and unexpectedly large difficulties acquiring Swedish, despite receiving ample input in Swedish. Thus, scoring well in minority-language Turkish tasks disguises the severe problems this child has in his majority language, Swedish.

BiTurLI-03 (age 5;11) had a similar profile to BiTurLI-01, as his Turkish vocabulary and narrative scores were all well below the −1.25 cut-off, but his Swedish scores (save for narrative production) were close to the age-group mean. He had received regular exposure to both Turkish and Swedish since birth, and had a relatively high proportion of daily exposure to Turkish, even at almost six years of age. Despite such ample Turkish input, both the parents and the bilingual Turkish/Swedish-speaking schoolteacher emphasized that BiLI-03′s Turkish was very limited, and that he always answered in Swedish when spoken to in Turkish. Also, the experimenter during the Turkish session reported that the child basically spoke no Turkish during assessment. Similarly to BiTurLI-01, the child’s scoring close to the mean in one language (majority language Swedish) ‘hides’ the severe problems the child has in his other language (minority language Turkish).

BiTurLI-04 (age 4;9) did not have a stronger language, as her vocabulary and narrative scores were all similarly low in both Turkish and Swedish. Exposure to Turkish had started at birth, and exposure to Swedish within the second year of life. Current daily exposure was slightly higher in Turkish (60%) than in Swedish (40%). Additionally, the parents, the preschool teachers, and the SLP reported that the child preferred to speak English. According to the parents, BiTurLI-04 spent a lot of time in front of screens, mostly watching YouTube videos in English. They were also of the opinion that their child did not receive enough ‘proper’ Swedish input at school. The teacher reported that BiTurLI-04 had very limited Swedish and had difficulties with social interaction, and that she was frequently involved in peer conflicts at school.

BiTurLI-05 (age 6;10) scored low in both languages, but particularly low in Swedish comprehension of narratives and vocabulary. This child had Kurdish as his first language, but was also exposed to Turkish and Swedish from an early age (Turkish from age 1, Swedish from age 2). By the time of testing, he had more exposure to Turkish and Swedish (estimated daily exposure was 40% for both languages) than Kurdish (estimated daily exposure was 20%). Thus, despite having similar proportions of exposure to Turkish and Swedish, scores were considerably lower in Swedish than in Turkish. The parents reported that he had a late language development, and that he rarely initiated communication outside the family, due to shyness. The teacher also described him as a quiet child who showed a slow development at school and often needed one-on-one instruction. Similarly to the parents and the teacher, BiTurLI-05′s SLP described him as a quiet boy with pragmatic difficulties and limited conceptual knowledge, who made slow progress in therapy.

Finally, BiTurLI-06 (age 8;1) had a similar profile to BiTurLI-04, who did not score clearly better in one language than the other. BiTurLI-06 was exposed to both Kurdish and Turkish from birth and began to receive regular exposure to Swedish at age three. By the time of testing (at eight years of age), she had proportionally more daily exposure to Swedish (50%) than to Turkish (30%) and Kurdish (20%). The parents originally stated that there were no problems in either language, but later gave contradictory information in the interview, saying that she had poor Swedish comprehension. The parents, the teacher, and the experimenter all described BiTurLI-06 as a social child. However, the teacher reported that she often had difficulties understanding instructions. She had a few friends at school but there were sometimes misunderstandings, which made BiTurLI-06 frustrated when she could not get her message across. According to the SLP, she had difficulties in all areas of language, and often misunderstood simple instructions.

These examples illustrate how individual DLD children can have similar language scores/profiles, despite very different exposure patterns, and they underscore the need for interpreting results from formal language assessment in relation to language exposure and in relation to descriptions and observations of functional language and communication skills in naturalistic contexts.

## 8. Conclusions and Future Directions

In line with Paradis et al. (2013 [33]) and Govindarajan and Paradis (2019 [32]), we conclude that language scores do not really tell so much on their own, and propose that results from language assessment tasks, even those developed specifically for bilinguals, such as the LITMUS tools, need to be interpreted in light of information about language exposure. This information about language exposure and use can be gathered from questionnaires and interviews with parents and teachers (Paradis et al., 2010 [43]; Tuller 2015 [42]). In addition, conducting interviews with parents and teachers in conjunction with formal assessment makes it possible to obtain a picture of the children’s functional language skills and can identify communication difficulties that are hard to catch with standardized tests (Öberg & Bohnacker 2022 [14]). We see that an increased use of mixed-methods research designs that combine quantitative and qualitative methods may be fruitful in the advancement of investigating language assessment in bilingual children, and in developing assessment methods that are valid for identifying DLD in bilingual populations (e.g., Glogowska 2011 [88]).

The DLD children in the present study generally had low vocabulary and narrative macrostructure comprehension scores in the home/minority language, despite extensive and sustained exposure to that language. Many also had comparably low vocabulary and narrative comprehension scores in the majority language, despite extensive exposure to the majority language. Whilst one should be cautious when making generalizations based on a DLD sample as limited in size as ours, low comprehension scores in a bilingual child, *despite* continuous, extensive exposure to that language from an early age, might be considered a warning sign for DLD. Recent advances suggest that the language skills of bilingual TD and DLD populations may be differently affected by language exposure and cognitive abilities, such as analytical reasoning and short-term memory (Paradis 2023 [89]), see also, Smolander et al. (2021 [60]) on input and vocabulary development in the L2, and Govindarajan and Paradis (2019 [32]) on input and narrative production in the L2. However, more research needs to be undertaken before the association between language exposure, cognition and language abilities in bilingual TD and DLD populations is well understood. Future work is therefore required to establish whether language scores of children with DLD are affected by background factors such as input, cognitive abilities and other language measures in the same way as for their TD peers, and whether these (non-) differences are dependent on the measure, i.e., the language domain, the modality (comprehension or production) and the minority or majority language.

## Figures and Tables

**Table 1:** Participants in the TD sample: proportion girls/boys, mean age and age range.

	4 Years (n = 27)	5 Years (n = 25)	6 Years (n = 27)	7 Years (n = 29)	Total (n = 108)
**Girls/boys**	14/13	15/10	15/12	14/15	58/50
**Mean age**	4;6	5;5	6;6	7;6	6;0
**Age range**	4;0–4;11	5:0–5;11	6;0–6;11	7;0–8;1 *	4;0–8;1 *

*Note.* * Two children in the 7-year-old group had just turned 8 at the time of testing.

**Table 2:** The participants in the DLD sample: age, age of onset of Turkish and Swedish, estimated daily exposure, (pre)school attendance and DLD diagnosis.

	Age	Age of Onset		Daily Exposure	(Pre)school	DLD Diagnosis
		Tur	Swe			
**BiTurLI-01**	6;3	at birth	1;0–1;11	Swe 80%, Tur 20%	Språkförskola	Mixed rec. and expr. LD
**BiTurLI-02**	5;4	at birth	2;0–2;11	Swe 40%, Tur 60%	Preschool	Mixed rec. and expr. LD + pragm.
**BiTurLI-03**	5;11	at birth	at birth	Swe 50%, Tur 50%	Preschool	Expr. LD (previously mixed rec. and expr. LD)
**BiTurLI-04**	4;9	at birth	1;0–1;11	Swe 40%, Tur 60%	Preschool	Mixed rec. and expr. LD + pragm.
**BiTurLI-05**	6;10	1;0–1;11	2;0–2;11	Swe 40%, Tur 40%, Kur 20%	Förskoleklass	Mixed rec. and expr. LD + pragm.
**BiTurLI-06**	8;1	at birth	3;0–3;11	Swe 50%, Tur 30%, Kur 20%	1st grade	Unspec. LD (likely mixed rec. and expr. LD)

*Note.* (D)LD = (Developmental) Language Disorder, rec. = receptive, expr. = expressive, pragm. = pragmatic difficulties, *Språkförskola* = a specialized preschool unit for children with severe DLD, *Förskoleklass* = a preparatory year between preschool and primary school. BiTurLI-05 was first exposed to Kurdish only (therefore, AoO was not at birth for either Tur or Swe).

**Table 3:** Number of children who completed each task (TD sample).

	4 Years	5 Years	6 Years	7 Years	Total
**NWR LS-Tur and CL-Tur**	N = 26	N = 25	N = 26	N = 26	N = 103
**Turkish vocabulary comp and prod**	N = 25	N = 23	N = 26	N = 28	N = 102
**Swedish vocabulary comp and prod**	N = 25	N = 23	N = 26	N = 28	N = 102
**Turkish Cat/Dog narrative comp/prod**	N = 24/24	N = 22/22	N = 26/26	N = 28/28	N = 100/100
**Swedish Cat/Dog narrative comp/prod**	N = 23/25	N = 22/23	N = 26/26	N = 28/28	N = 99/102
**Turkish BB/BG narrative comp/prod**	N = 24/24	N = 21/22	N = 26/26	N = 28/28	N = 99/100
**Swedish BB/BG narrative comp/prod**	N = 23/24	N = 22/22	N = 26/26	N = 28/28	N = 99/100

*Note.* One five-year-old only completed the Turkish session. One seven-year-old only completed the Swedish session.

**Table 4:** Mean scores, standard deviations (SD) and ranges for the NWR tasks by age groups. Max score = 16 for both tasks.

		4 Years	5 Years	6 Years	7 Years	Total
**LS-Tur**	Mean (SD)	9.6 (3.1)	10.6 (2.5)	11.1 (2.6)	11.6 (2.4)	10.7 (2.7)
	Range	4–14	6–15	4–15	5–16	4–16
**CL-Tur**	Mean (SD)	10.9 (2.3)	11.2 (2.2)	11.7 (2.8)	12.6 (1.8)	11.6 (2.4)
	Range	7–15	7–15	4–16	8–15	4–16

**Table 5:** Mean scores, standard deviations (SDs) and ranges for Turkish and Swedish CLT vocabulary comprehension and production by age groups. Max score = 60 for all tasks.

		4 Years	5 Years	6 Years	7 Years	Total
**Turkish vocabulary** **comprehension**	Mean (SD)	51.4 (5.7)	53.8 (8.3)	54.4 (7.1)	56.9 (4.1)	54.2 (6.6)
	Range	35–58	26–60	27–60	40–60	26–60
**Turkish vocabulary** **production**	Mean (SD)	36.4 (10.3)	37.6 (13.2)	39.5 (12.2)	42.4 (10.9)	39.1 (11.7)
	Range	5–52	3–56	12–53	13–60	3–60
**Swedish vocabulary comprehension**	Mean (SD)	41.2 (9.3)	48.6 (8.1)	55.3 (4.5)	54.9 (5.0)	50.2 (9.0)
	Range	18–60	31–59	45–60	42–60	18–60
**Swedish vocabulary production**	Mean (SD)	25.4 (9.6)	31.1 (9.2)	39.8 (7.7)	42.1 (9.0)	34.9 (11.1)
	Range	8–47	16–46	25–51	19–54	8–54

**Table 6:** Mean scores, standard deviations (SDs) and ranges for Turkish and Swedish narrative macrostructure comprehension (max score = 20) and production (max score = 34) by age groups. Cat/Dog and Baby Birds/Baby Goats are combined.

		4 Years	5 Years	6 Years	7 Years	Total
**Turkish narrative** **comprehension**	Mean (SD)	9.8 (4.5)	12.4 (5.5)	14.1 (4.3)	15.1 (3.9)	12.9 (4.9)
	Range	0–17	0–19	1–20	1–20	0–20
**Turkish narrative** **production**	Mean (SD)	6.2 (3.9)	8.7 (4.5)	10.2 (3.5)	12.5 (3.7)	9.5 (4.5)
	Range	0–11	0–15	3–16	1–18	0–18
**Swedish narrative comprehension**	Mean (SD)	8.8 (5.0)	11.9 (4.9)	15.7 (1.8)	17.2 (2.1)	13.6 (4.9)
	Range	0–16	0–19	12–19	10–20	0–20
**Swedish narrative production**	Mean (SD)	6.0 (4.2)	8.9 (3.8)	11.5 (3.0)	13.7 (3.4)	10.1 (4.6)
	Range	0–15	3–17	7–17	5–20	0–20

**Table 7:** NWR, vocabulary and narrative macrostructure scores (raw scores and age-adjusted z-scores) for the children in the DLD sample.

			NWR		Vocabulary				Narrative Macrostructure			
	Age	Score	LS-Tur	CL-Tur	Turkish		Swedish		Turkish		Swedish	
					Comp	Prod	Comp	Prod	Comp	Prod	Comp	Prod
**BiTur** **LI-01**	6;3	raw	4	2	30	4	57	34	1	0	15	16
		z	−2.76 *	−2.71 *	−3.43 *	−2.92 *	0.38	−0.75	−3.02 *	−2.90 *	−0.40	1.53
**BiTur** **LI-02**	5;4	raw	3	5	59	38	35	15	13	9	9	5
		z	−2.98 *	−2.81 *	0.63	0.03	−1.67 *	−1.75 *	0.11	0.08	−0.60	−1.03
**BiTur** **LI-03**	5;11	raw	7	14	24	5	46	32	0	0	10	0
		z	−1.40 *	1.24	−3.59 *	−2.46 *	−0.32	0.10	−2.24 *	−1.91 *	−0.39	−2.34 *
**BiTur** **LI-04**	4;9	raw	10	14	40	25	24	15	3	3	3	3
		z	0.12	1.37	−2.00 *	−1.11	−1.85 *	−1.08	−1.53 *	−0.83	−1.16	−0.71
**BiTur** **LI-05**	6;10	raw	3	9	45	21	25	12	3	5	3	1
		z	−3.14 *	-0.94	−1.33 *	−1.52 *	−6.75 *	−3.61 *	−2.56 *	−1.48 *	−6.92 *	−3.52 *
**BiTur** **LI-06**	8;1	raw	11	11	49	38	47	33	8	8	10	13
		z	−0.25	−0.89	−1.90 *	−0.41	−1.58 *	−1.00	−1.83 *	−1.20	−3.41 *	−0.20

*Note.* Z-scores < −1.25 are in bold and marked with *. BiTurLI-06 is compared to the group closest in age (the 7-year-olds).

## Data Availability

The present study is part of a larger, ongoing research project on the multilingual development of Turkish/Swedish and Arabic/Swedish. The original raw dataset cannot be shared in full, as this would breach participant confidentiality and GDPR guidance. For data supporting the reported results, please consult the authors.

## Notes

1 DLD (Developmental language disorder), also known as (Specific) language impairment (SLI) or Primary language impairment, manifests as a pronounced deficit in the development of language skills, which cannot be attributed to intellectual disability, hearing impairment, medical syndromes or neurological disorders (Bishop 1997, pp. 21–23 [2]; Leonard 2014, p. 3 [3]). It negatively affects children’s oral communication, literacy and advancement in school (Norbury et al., 2016 [4]). DLD is a heterogeneous disorder where deficits in a particular language domain may be more pronounced for some individuals than for others.

2 Nonword repetition and learning new words both require the ability to temporarily store phonological representations. The phonological loop enables individuals to quietly rehearse linguistic material that is otherwise subject to rapid decay in short-term memory, and is therefore thought to play an important role particularly in the early stages of language acquisition (Gathercole 2006 [7]).

3 When suspecting DLD, it is otherwise recommended that *both* languages of a bilingual child be assessed (Kohnert 2010 [24]; Peña, Bedore & Kester 2016 [25].

4 In addition, several papers (e.g., Abed Ibrahim and Hamann 2017 [54]; Hamann and Abed Ibrahim 2017 [55]; de Almeida et al., 2017 [56]; and Tuller et al., 2018 [57]) report on NWR in subsamples of the same participant groups as Chilla et al. (2021 [52]).

5 Moreover, Öberg (2020 [58]) compared these children’s performance on near-identical versions of the language-independent NWR task (2-to-5 syllables), once administered in Swedish and once administered in Arabic (with slightly different phoneme realizations). Performance was similar but slightly depressed for the Arabic version, mainly due to some item effects.

6 Khoury et al. (2017 [61]) chose to implement a conceptual vocabulary scoring procedure for CLT production (expressive vocabulary) because of widespread code-mixing in Lebanese society and in the children’s immediate environment. Thus, Lebanese Arabic, French, and English words corresponding to the CLT target items were scored as correct on the CLT-LB (i.e., Lebanese Arabic) picture naming task.

7 Khoury et al. (2017 [61]) also mentioned that certain types of incorrect CLT responses were more frequent in the DLD than the TD group, such as *I don’t know*, no response, onomatopoetic answers, and gestures.

8 MAIN includes four picture-based fictional stories carefully designed to be comparable in length and macrostructural components (see Section 4.2.3 and Section 4.3), standardized prompts, comprehension questions and scoring protocols for macrostructure (for details see Gagarina et al., 2012 [68], 2019 [41]; Bohnacker & Gagarina 2020a [69]). MAIN can be used to assess story generation (‘telling’), story retelling (after listening), and story comprehension (where the child is probed to make inferences from the pictures and verbalize this understanding).

9 Recall that Boerma et al.’s DLD sample consisted of children in special education who performed at least 2 SD below the mean on a standardized Dutch language test or at least 1.5 SD below the mean on two subscales. This preselection might have enlarged the difference between the DLD and TD samples.

10 As mentioned in Section 2.2 and Section 2.3, for 108 of these participants, some vocabulary and macrostructure results have been reported previously in a doctoral thesis (Öztekin 2019 [62]). For the present study, the dataset has been considerably expanded and it is explored from different angles and with multivariate statistics.

11 One child was accidentally seen twice, once at age 4 (when testing was terminated early) and again one year later at age 5 (testing sessions completed). Only the data at age 5 were included in the study.

12 One child, BiTurLI-03, was 5;11 at the first testing (in Turkish), but had turned 6 by the second testing. His scores are compared to the five-year-olds in the TD sample.

13 Another vowel harmony rule only applies to high vowels, making them agree in rounding with the preceding vowels. In many lexical borrowings, however, vowel harmony does not apply.

14 According to a large-scale norming study, monolingual TD children generally master Turkish phonology early, i.e., before age 3;6 (Topbaş 1997 [73], 2006 [74]). The norming study also provides reference data for the typical age of acquisition of individual phonemes.

15 In a few cases, headphones were not used, and the audio was played from the laptop speakers.

16 For neither NWR task was there any correlation with language exposure or SES (parental education.

17 A model with Turkish vocabulary production yielded equivalent results.

18 A model with Turkish vocabulary comprehension yielded equivalent results.

19 A model with Swedish vocabulary comprehension yielded equivalent results.

20 Note that this particular child was described by both parents, the teacher, and the SLP as having severe communication difficulties (see Table A1 in the Appendix A). The experimenter in the Swedish session also reported that the child often repeated the experimenter’s utterances, seemingly without understanding their meaning. This ‘echoing’ behavior could provide an explanation for BiTurLI-04’s above-average performance on both NWR tasks.

21 The same pattern was found for the Turkish comprehension scores.

22 This also held for the Swedish comprehension scores.

## Appendix A

**Table A1:** Language abilities and behavior of the DLD children as described by parents, teachers, SLPs and experimenters.

		Parents	Teacher	SLP	Experimenters’ Observations
**BiTur** **LI-01**	*language*	Tur: −comp, −prod Swe: so-so	Swe: −comp, −prod	−comp, −prod, −vocab, −perspective taking	Swe: −comp Tur: −comp
	*behavior/comments*	LLD; Tur is very limited; answers in Swe when spoken to in Tur	engages in communication, but frequent misunderstandings/conflicts; uses a lot of Eng	gets upset about small things, wants things her own way; cooperates in therapy, but progress is slow; uses a lot of Eng	Swe: interacts with Exp and the other children at preschool; cooperates well during assessment Tur: misunderstands instructions
**BiTur** **LI-02**	*language*	Tur: +comp, +prod Swe: so-so comp, −prod	Swe: −comp, −prod	−comp, −prod, difficulties with word learning	Swe: −prod
	*behavior/comments*	LLD in Swe; Swe is very limited; had hearing problems in the past	seeks help from Tur-speaking peers when he does not know a word in Swe; speaks Tur with non-Tur-speakers; has trouble ‘remembering’ info in Swe	Swe virtually ‘non-existent’, has great difficulties learning/consolidating new words in Swe; speaks Tur to non-Tur speakers; attention difficulties	Swe: child is difficult to understand
**BiTur** **LI-03**	*language*	Tur: +comp, −prod Swe: +comp, +prod	Swe: −prod, −vocabulary, −conversational turn-taking Tur: comp ok	−comp, −prod, −word finding	Tur: −prod
	*behavior/comments*	LLD in Tur; had hearing problems until age 3; always answers in Swe when spoken to in Tur	polite and ‘nice’ child; language skills have developed a lot; functions well in the group; can follow instructions; Tur is limited acc to Tur-speaking teacher	has trouble making himself understood; cooperates well in therapy, but has attention difficulties	Swe: social and positive at preschool; interacts with the other children Tur: basically speaks no Tur
**BiTur** **LI-04**	*language*	Tur: +comp, +prod Swe: +comp, −prod Eng better than Swe	Swe: −comp, −prod	−comp	Swe: −comp, −prod
	*behavior/comments*	not enough ‘proper’ Swe input at school; prefers to speak Eng, spends a lot of time with digital devices in Eng	prefers Eng; very limited Swe; difficulties with social interaction; has jealousy issues and obsessions, frequent peer conflicts; needs one-on-one instructions	very limited Swe; pragmatic difficulties; needs repeated instructions; has attention difficulties; SLP thinks a psychological assessment would be advisable	Swe: talks in a shrill voice and uses many phrases in Eng that seem overheard from Youtube videos (not in a communicative way); repeats what Exp says without understanding what it means; needs a lot of support to complete assessment
**BiTur** **LI-05**	*language*	Tur: −comp, −prod Swe: +comp, +prod	Swe: −comp, −prod	−comp, −prod, −vocab	Swe: −comp, −prod
	*behavior/comments*	LLD; rarely initiates communication outside the family, is shy	often quiet; rarely shows facial expressions; needs one-on-one instructions; slow development, teacher thinks child would be better off in a smaller group/class with more tailored support	pragmatic difficulties; often quiet, slow progress in therapy; limited conceptual knowledge	Swe and Tur: cooperative during testing at home and at preschool; speaks in short utterances in both languages
**BiTur** **LI-06**	*language*	Tur: +comp, +prod (‘very good’) Swe: +comp, +prod, but contradictory info in interview: poor Swe comp	Swe: −comp, −prod	−comp, −prod	Swe: −prod
	*behavior/comments*	LLD; social, likes to play with friends; only problems at school, no problems at home	has trouble understanding instructions; becomes angry when she cannot make herself understood; has a few friends but there are sometimes misunderstandings	has difficulties in all areas of language, misunderstands simple questions; SLP has not been able to conduct an assessment in Tur, since the parents think that the child only has difficulties in Swe	Swe: social and interacts with other children at school, other children seem to like her; cooperates well during assessment but is easily distracted

*Note*. LLD = Late language development; ‘comp’ = language comprehension; ‘prod’ = language production; ‘−’ indicates relative weaknesses, ‘+’ indicates relative strengths in language ability.

**Table A2:** All items in the NWR tasks: LS-Tur (language-specific Turkish), CL-Tur (cross-linguistic Turkish), LS-Swe (language-specific Swedish) and CL-Tur (cross-linguistic Swedish).

**LS-Tur**	/tekyn/ /pajɯz/ /dʒɛlit/ /kølin/ /disɛlyk/ /konamɯʃ/ /ɡakosɯɫ/ /petylis/ /tediɡølyk/ /søɡynydɛn/ /joʃaɡɯmut/ /dʒɯnojuɫam/ /matɯsadʒɯjon/ /ɡedisedʒimiz/ /liɡømydʒyʋɛn/ /tɯjɯboɡuɫas/
**CL-Tur**	/zibu/ /duɫa/ /naɡi/ /ɫumi/ /sipuɫa/ /bamudi/ /malitu/ /ɫumiɡa/ /zipaleda/ /mukitaɫɯ/ /kazuɫumi/ /lidisakɯ/ /sipumakelɯ/ /tulikezuma/ /maɫuziɡubɯ/ /litɯpimuti/
**CL-Swe**	/sɪbʊ/ /dʊla/ /naɡɪ/ /lʊnɪ/ /sɪpʊla/ /banʊdɪ/ /malɪtʊ/ /lɪmɪka/ /sɪbalɪta/ /mʊkɪdala/ /ɡasʊlʊmɪ/ /lɪdɪsakʊ/ /sɪpʊnakɪla/ /tʊlɪɡasʊmʊ/ /malʊsɪɡʊba/ /lɪdapɪmʊtɪ/
**LS-Swe**	/ɡlʏˈvoː/ /aˈpɛt/ /ɪˈfʉːm/ /ˈɧɔɾjɛ/ /naˈkiːt/ /ˈspʉːmɛ/ /lɛbʊˈsʉːf/ /mɵstɾɛˈfalj / /ɡlɛŋɛˈsɵlp/ /salʊˈtɑːn/ /hœntˈpʉ:lɛ/ /nɛsʊˈloː/ /ˈmaŋɛʂˌblɛɡɛ/ /ɛlʊˈmɔkɪ/ /ɔlɪˈtʉːkɛ/ /spɵɾɪfɾaˈɡoːl/ /tɪbɛˈfiːmɛ/ /lɵtʊspɛˈlʉːn/ /tœlɪpaˈleːɾʊ/ /ɕɵlɛˌkɾɔmpaˈmiːd/ /fɪmɪɡlaˈnɛftɪ/ /hɪlʊteɾaˈpʉːd/ /flɛtɛˌmɪŋɛˈɾoːf/ /dalabɛlˈhiːmɛ/

**Table A3:** Summary of logistic mixed-effects regression model (Model 1): accuracy of CL-Tur vs. LS-Tur task.

*Random Effects*	Variance	SD	*Fixed Effects*		B	SE (B)		z	*p*
Participant	0.44	0.67	Intercept		1.42 ***	0.23		6.14	<0.001
Item	0.68	0.82	Age		0.20 *	0.09		2.37	0.02
			Turkish vocabulary		−0.05	0.10		−0.46	0.64
			Task (CL-Tur vs. LS-Tur)		−0.42	0.31		−1.36	0.17
			Syllables		−0.82 ***	0.16		−5.31	<0.001
			Task (CL-Tur) × Tur vocab		0.21 *	0.09		2.30	0.02
*Model evaluation*	**Marginal R^2^** 0.15			**Conditional R^2^** 0.37			**C-index** 0.82		

*Note.* Logistic mixed-effects regression model with random effects: random intercepts for participant and test item. Model fit with maximum likelihood (Laplace approximation). The reference level for categorical variables is the first category. *** *p* < 0.001, * *p* < 0.05.

**Table A4:** Summary of linear regression models: Turkish vocabulary comprehension and production.

	Model 2 (Turkish Vocabulary Comprehension)					Model 3 (Turkish Vocabulary Production)				
	B	SE (B)	t	*p*	β	B	SE (B)	t	*p*	β
Intercept	54.49 ***	0.54	100.74	<0.001	–	39.38 ***	1.03	38.41	<0.001	–
Age	0.21 ***	0.05	4.37	<0.001	0.50	0.36 ***	0.09	3.91	<0.001	0.44
LoE Swe	−0.07	0.04	−1.92	0.057	−0.22	−0.19 **	0.07	−2.86	0.005	−0.33
Daily exp Tur	0.07 **	0.03	2.67	0.009	0.25	0.15 **	0.05	2.82	0.005	0.26
	R^2^ (adj.) = 0.20, *F*(3,97) = 9.13, *p* < 0.001					R^2^ (adj.) = 0.19, *F*(3,97) = 8.83, *p* < 0.001				

*Note.* VIF values were 1–2 for all predictors, indicating low levels of collinearity. *** *p* < 0.001, ** *p* < 0.01.

**Table A5:** Summary of linear regression models: Swedish vocabulary comprehension and production.

	Model 4 (Swedish Vocabulary Comprehension)					Model 5 (Swedish Vocabulary Production)				
	B	SE (B)	t	*p*	β	B	SE (B)	t	*p*	β
Intercept	50.17 ***	0.64	78.17	<0.001	–	34.82 ***	0.76	45.90	<0.001	–
Age	0.24 ***	0.06	4.22	<0.001	0.38	0.28 ***	0.07	4.12	<0.001	0.36
LoE Swe	0.16 ***	0.04	3.68	<0.001	0.34	0.19 ***	0.05	3.83	<0.001	0.34
Daily exp Swe	0.07 *	0.03	2.15	0.03	0.16	0.14 ***	0.04	3.63	<0.001	0.26
	R^2^ (adj.) = 0.49, *F*(3,97) = 32.40, *p* < 0.001					R^2^ (adj.) = 0.53, *F*(3,97) = 38.08, *p* < 0.001				

*Note.* VIF values were 1–2 for all predictors, indicating low levels of collinearity. *** *p* < 0.001, * *p* < 0.05.

**Table A6:** Summary of linear regression models: Turkish narrative macrostructure comprehension and production.

	Model 6 (Turkish Narrative Comprehension)					Model 7 (Turkish Narrative Production)				
	B	SE (B)	t	*p*	β	B	SE (B)	t	*p*	β
Intercept	12.97 ***	0.34	38.42	<0.001	–	9.50 ***	0.30	31.98	<0.001	–
Age	0.09 **	0.03	2.88	0.004	0.27	0.12 ***	0.03	4.04	<0.001	0.36
LoE Swe	0.01	0.02	0.60	0.55	0.06	0.03	0.02	1.38	0.17	0.12
Daily exp Tur	0.00	0.02	0.22	0.82	0.02	0.01	0.02	0.50	0.61	0.04
Tur vocabulary	0.27 ***	0.03	8.05	<0.001	0.62	0.22 ***	0.03	7.48	<0.001	0.56
	R^2^ (adj.) = 0.53, *F*(4,96) = 29.05, *p* < 0.001					R^2^ (adj.) = 0.57, *F*(4,96) = 33.54, *p* < 0.001				

*Note.* ‘Tur vocabulary’ denotes vocabulary production scores. VIF values were 1–2 for all predictors, indicating low levels of collinearity. *** *p* < 0.001, ** *p* < 0.01.

**Table A7:** Summary of linear regression models: Swedish narrative macrostructure comprehension and production.

	Model 8 (Swedish Narrative Comprehension)					Model 9 (Swedish Narrative Production)				
	B	SE (B)	t	*p*	β	B	SE (B)	t	*p*	β
Intercept	13.56 ***	0.29	46.14	<0.001	–	10.09 ***	0.29	35.23	<0.001	–
Age	0.13 ***	0.03	4.37	<0.001	0.36	0.11 ***	0.03	3.77	<0.001	0.32
LoE Swe	−0.01	0.02	−0.67	0.51	−0.06	0.00	0.02	0.01	0.99	0.00
Daily exp Swe	−0.01	0.02	−0.91	0.36	−0.06	0.00	0.02	−0.05	0.96	0.00
Swe vocabulary	0.27 ***	0.04	6.81	<0.001	0.60	0.23 ***	0.04	5.92	<0.001	0.55
	R^2^ (adj.) = 0.64, *F*(4,96) = 45.79, *p* < 0.001					R^2^ (adj.) = 0.61, *F*(4,96) = 39.33, *p* < 0.001				

*Note.* ‘Swe vocabulary’ denotes vocabulary production scores. VIF values were 1–2 for all predictors, indicating low levels of collinearity. *** *p* < 0.001.

**Table A8:** Mean scores, standard deviations (SDs) and ranges for Turkish and Swedish narrative macrostructure comprehension (max score = 10) and story structure production (max score = 17) by age group (MAIN Cat/Dog).

		4 Years	5 Years	6 Years	7 Years	Total
**Turkish narrative** **comprehension**	Mean (SD)	5.8 (2.2)	7.0 (2.7)	7.8 (2.2)	7.9 (2.0)	7.2 (2.4)
	Range	2–10	0–10	1–10	1–10	0–10
**Turkish narrative** **production**	Mean (SD)	3.2 (1.8)	4.3 (2.4)	4.6 (1.7)	6.2 (2.1)	4.6 (2.3)
	Range	0–6	1–10	1–7	0–9	0–10
**Swedish narrative comprehension**	Mean (SD)	6.0 (2.6)	7.1 (2.3)	8.6 (1.1)	8.8 (1.3)	7.7 (2.2)
	Range	1–9	1–10	6–10	6–10	1–10
**Swedish narrative production**	Mean (SD)	2.8 (1.7)	4.7 (1.9)	5.9 (1.7)	7.1 (2.1)	5.2 (2.5)
	Range	0–6	1–8	2–9	2–12	0–12

**Table A9:** Mean scores, standard deviations (SDs) and ranges for Turkish and Swedish narrative macrostructure comprehension (max score = 10) and story structure production (max score = 17) by age group (MAIN Baby Birds/Baby Goats).

		4 Years	5 Years	6 Years	7 Years	Total
**Turkish narrative** **comprehension**	Mean (SD)	4.4 (2.4)	6.2 (2.4)	6.3 (2.5)	7.2 (2.5)	6.1 (2.6)
	Range	0–9	0–9	1–10	0–10	0–10
**Turkish narrative** **production**	Mean (SD)	3.3 (2.6)	4.7 (2.7)	5.6 (2.4)	6.3 (2.4)	5.1 (2.7)
	Range	0–8	0–9	1–9	1–11	0–11
**Swedish narrative comprehension**	Mean (SD)	3.6 (2.3)	5.3 (2.3)	7.1 (1.7)	8.4 (1.4)	6.2 (2.6)
	Range	0–8	0–10	3–10	4–10	0–10
**Swedish narrative production**	Mean (SD)	3.3 (2.8)	4.4 (2.7)	5.6 (2.1)	6.5 (2.1)	5.0 (2.7)
	Range	0–9	0–9	1–10	1–11	0–11

**Table A10:** Individual NWR raw scores and percentages for the DLD children.

	Age	LS-Tur	CL-Tur	CL-Swe	LS-Swe
**BiTurLI-01**	6;3	4 (25%)	4 (25%)	6 (38%)	6 (25%)
**BiTurLI-02**	5;4	3 (19%)	5 (31%)	7 (44%)	6 (25%)
**BiTurLI-03**	5;11	7 (44%)	14 (88%)	13 (81%)	12 (50%)
**BiTurLI-04**	4;9	10 (63%)	14 (88%)	11 (69%)	14 (58%)
**BiTurLI-05**	6;10	3 (19%)	9 (56%)	8 (50%)	9 (38%)
**BiTurLI-06**	8;1	11 (69%)	11 (69%)	10 (63%)	7 (29%)

*Note.* LS-Tur = Language-Specific Turkish task (max score = 16), CL-Tur = Cross-Linguistic Turkish task (max score = 16), CL-Swe = Cross-Linguistic Swedish task (max score = 16), LS-Swe = Language-Specific Swedish task (max score = 24).
